# The Serum and Saliva Proteome of Dogs with Diabetes Mellitus

**DOI:** 10.3390/ani10122261

**Published:** 2020-12-01

**Authors:** Lorena Franco-Martínez, Andrea Gelemanović, Anita Horvatić, María Dolores Contreras-Aguilar, Vladimir Mrljak, José Joaquín Cerón, Silvia Martínez-Subiela, Asta Tvarijonaviciute

**Affiliations:** 1 Interdisciplinary Laboratory of Clinical Pathology, Interlab-UMU, Regional Campus of International Excellence ‘Campus Mare Nostrum’, University of Murcia, 30100 Murcia, Spain; lorena.franco2@um.es (L.F.-M.); mariadolores.contreras@um.es (M.D.C.-A.); jjceron@um.es (J.J.C.); asta@um.es (A.T.); 2 Mediterranean Institute for Life Sciences (MedILS), 21 000 Split, Croatia; agelemanovic@medils.hr; 3 Faculty of Food Technology and Biotechnology, University of Zagreb, Pierottijeva 6, 10 000 Zagreb, Croatia; horvatic.ani@gmail.com; 4 Faculty of Veterinary Medicine, University of Zagreb, Heinzelova 55, 10 000 Zagreb, Croatia; vmrljak@vef.hr

**Keywords:** canine diabetes mellitus, gel-free proteomics, high-resolution mass spectrometry, tandem mass tags, type 1 diabetes

## Abstract

**Simple Summary:**

The present study describes for the first time the differences in the serum and saliva proteomes between healthy dogs and dogs with diabetes mellitus by a high- throughput proteomic approach. More than 1000 proteins were identified, and 16 proteins in serum and 26 in saliva showed significant changes between both groups. Additionally, pathways that showed changes were discussed in order to improve the understanding of the pathophysiology of the disease and one protein in serum (haptoglobin) was successfully verified. The results of the present study could be a source of potential biomarkers for canine diabetes mellitus in saliva and serum and also contribute to increase the knowledge of the pathophysiology of the disease.

**Abstract:**

This study aims to evaluate the changes in salivary and serum proteomes that occur in canine diabetes mellitus type-1 (DM) through a high-throughput quantitative proteomic analysis. The proteomes of 10 paired serum and saliva samples from healthy controls (HC group, *n* = 5) and dogs with untreated DM (DM group, *n* = 5) were analyzed using Tandem Mass Tags (TMT)-based proteomic approach. Additionally, 24 serum samples from healthy controls and untreated DM were used to validate haptoglobin in serum. The TMT analysis quantified 767 and 389 proteins in saliva and serum, respectively. Of those, 16 unique proteins in serum and 26 in saliva were differently represented between DM and HC groups. The verification of haptoglobin in serum was in concordance with the proteomic data. Our results pointed out changes in both saliva and serum proteomes that reflect different physiopathological changes in dogs with DM. Although some of the proteins identified here, such as malate dehydrogenase or glyceraldehyde-3-phosphate dehydrogenase, were previously related with DM in dogs, most of the proteins modulated in serum and saliva are described in canine DM for the first time and could be a source of potential biomarkers of the disease. Additionally, the molecular function, biological process, pathways and protein class of the differential proteins were revealed, which could improve the understanding of the disease’s pathological mechanisms.

## 1. Background

Canine diabetes mellitus (DM) is a common spontaneous endocrine disorder in middle age to geriatric dogs [1]. Its estimated prevalence is between 0.2 to 1.2%, being higher in genetically predisposed breeds such as Cairn Terriers, Tibetan Terriers or Samoyeds [2]. According to data reported by Banfield’s State of Pet Health [3], canine DM showed a 79.7% increase in its incidence from 2006 to 2019 in the United States, affecting more than 2.5 million canine patients worldwide (Benfield’s State of Pet Health, 2016 Report). Canine DM has been regarded as similar to human type 1 DM, which is related to β-cell damage and insulin deficiency [4] and dogs have been proposed as feasible animal models for human DM [5].

DM is characterized by a persistent hyperglycemia and insulin deficiency, consequence of a massive pancreatic β-cell loss. These factors lead to polyuria, polydipsia, polyphagia, lethargy and weight loss [6]. The most common treatment for canine diabetes mellitus is the life-long subcutaneous injections of insulin twice a day. In untreated animals or when the disease cannot be adequately controlled, it can lead to potentially life-threatening diabetic ketoacidosis [7]. Additionally, there are common comorbidities and complications of canine DM, such as retinopathy, cataracts, hyperadrenocorticism, pancreatitis, dermatitis, otitis, urinary tract infections or hypothyroidism [7].

Saliva is a valuable body fluid that can reflect systemic and local health-conditions. Compared to blood, saliva collection is a non-invasive procedure that is easier, safer, less stressful and pain-free [8]. In humans changes in the proteome of saliva have been used to identify potential biomarkers of a variety of diseases such as cancer [9]. Also some reports indicate that saliva has a diagnostic and monitoring value in DM [10,11,12], and changes caused by diabetes mellitus in serum [13] and saliva [14,15,16] proteomes in humans have been reported. In dogs, gel-free proteomics approaches, including Tandem Mass Tag (TMT), have been successfully used in serum and/or saliva to study changes associated to obesity [17] or infectious diseases such as parvoviral enteritis [18] and leishmaniosis [19,20,21]. However, to the best of the author’s knowledge, there are no reports about studies addressing possible changes in saliva and serum proteomes in dogs with DM.

The aims of the present study were to detect the possible changes in paired saliva and serum proteomes in dogs with DM in comparison to healthy ones by using TMT-based technology. Those changes could contribute to gain knowledge of DM pathogenesis and the discovery of potential novel biomarkers of the disease.

## 2. Results

### 2.1. Proteomic Analysis in Serum

A total of 389 serum proteins remained for statistical analysis after removing of missing data, outliers and proteins with less of two unique peptides (Supplementary Materials Table S3 and Figure S1). Wilcoxon-Mann-Whitney test identified 31 proteins (corresponding to 16 unique genes) with differential abundances between HC and DM groups with *p*-value < 0.05, which are summarized after the removal of duplicates and isoforms in Table 1 and Figure S2.

**Figure 1:**
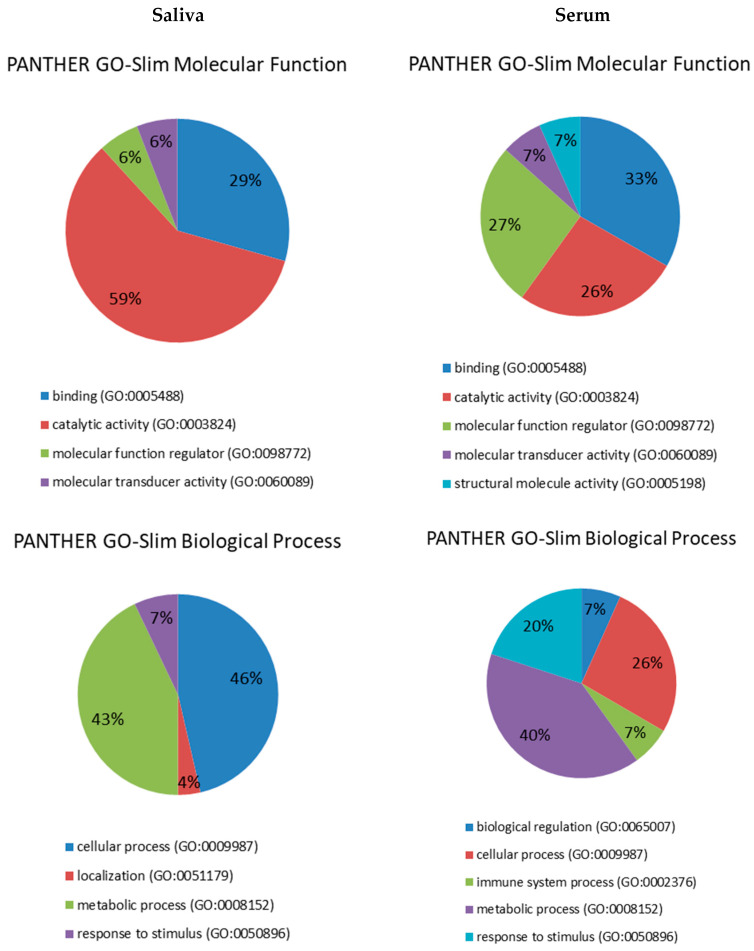

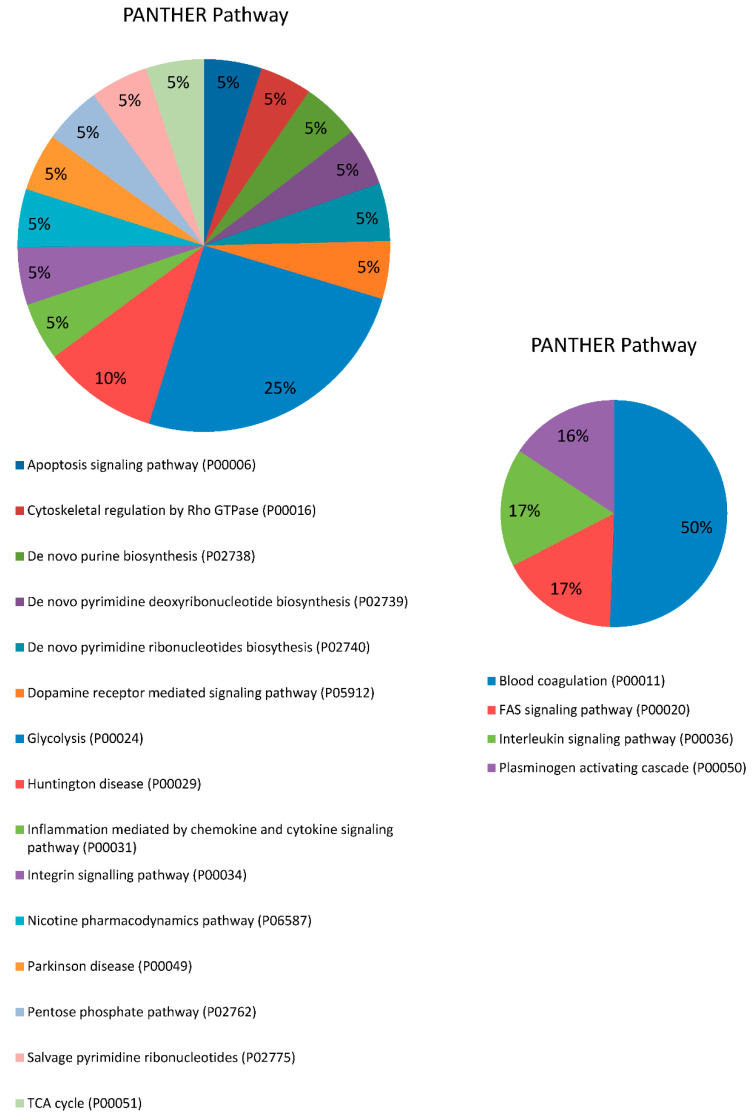

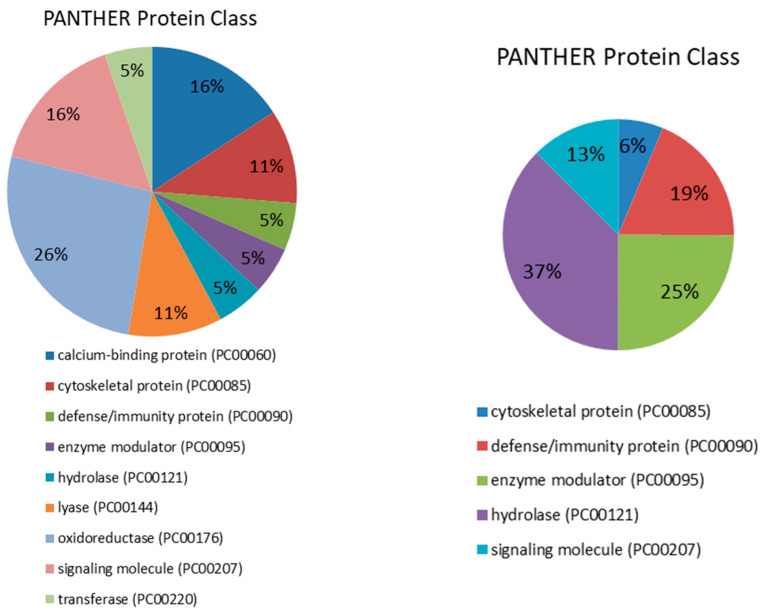
Pie charts showing the molecular function, biological process, pathway and protein class expressed as a percentage of the total differentially expressed proteins between HC and DM groups in serum (**right**) and saliva (**left**) based on the PANTHER classification system (http://www.pantherdb.org).

Of the 16 unique proteins differently modulated between HC and DM groups, 13 were down-regulated and three were up-regulated in DM. The proteins differently expressed in serum between HC and DM groups were used for subsequent bioinformatics analyses in terms of functional clusters, according to the PANTHER classification system, as shown in Figure 1.

Proteins were distributed within five different molecular functions: binding (GO:0005488) (33.3%), catalytic activity (GO:0003824) (26.7%), molecular function regulator (GO:0098772) (26.7%), molecular transducer activity (GO:0060089) (6.7%) and structural molecule activity (GO:0005198) (6.7%). They participate in five molecular processes, namely metabolic process (GO:0008152) (40%), cellular process (GO:0009987) (26.7%), response to stimulus (GO:0050896) (20%), immune system process (GO:0002376) (6.7% pl) and biological regulation (GO:0065007) (6.7%). Four pathways were found to be related to those differentially expressed proteins: blood coagulation (P00011) (42.9%), FAS signaling pathway (P00020) (14.3%), interleukin signaling pathway (P00036) (14.3%) and plasminogen activating cascade (P00050) (13.3%). Lastly, five proteins classes were identified, being hydrolase (PC00121) the most represented (37.5%), followed by enzyme modulator (PC00095) (25%), defense/immunity protein (PC00090) (18.7%), signaling molecule (PC00207) (12.5%) and cytoskeletal protein (PC00085) (6.3%).

### 2.2. Proteomic Analysis in Saliva

After removing missing data, outliers, not more than <5% FDR and proteins with less of two unique peptides, 767 proteins remained for statistical analysis (Table S4 and Figure S3). Wilcoxon-Mann-Whitney test identified 50 proteins (corresponding to 26 unique genes) with differential abundances between HC and DM groups with *p*-value < 0.05, which are summarized after the removal of duplicates and isoforms in Table 2 and Figure S4. Out of the 26 unique proteins modulated in saliva, five proteins were down-regulated in dogs with DM, while twenty-one were up-regulated.

These differentially expressed proteins in saliva between HC and DM groups were used for subsequent bioinformatics analysis in terms of functional clusters, according to the PANTHER classification system, as shown in Figure 1. Proteins were distributed within four different molecular functions, namely catalytic activity (GO:0003824) (58.8%), binding (GO:0005488) (29.4%), molecular function regulator (GO:0098772) (5.9%) and molecular transducer activity (GO:0060089) (5.9%). These proteins were involved in four molecular processes: cellular process (GO:0009987) (46.4%), metabolic process (GO:0008152) (42.9%), response to stimulus (GO:0050896) (7.1%) and localization (GO:0051179) (3.6%). There was identified a total of 15 pathways related to those differentially expressed proteins, being glycolysis (P00024) the most represented with 25%. Finally, nine protein classes were identified, being oxidoreductase (PC00176) the most represented (26.3%), followed by calcium-binding protein (PC00060) and signaling molecule (PC00207) (15.8% each).

No proteins were found to be differentially modulated between HC and DM groups in both serum and saliva.

### 2.3. Validation of Proteomic Results

**Figure 2:**
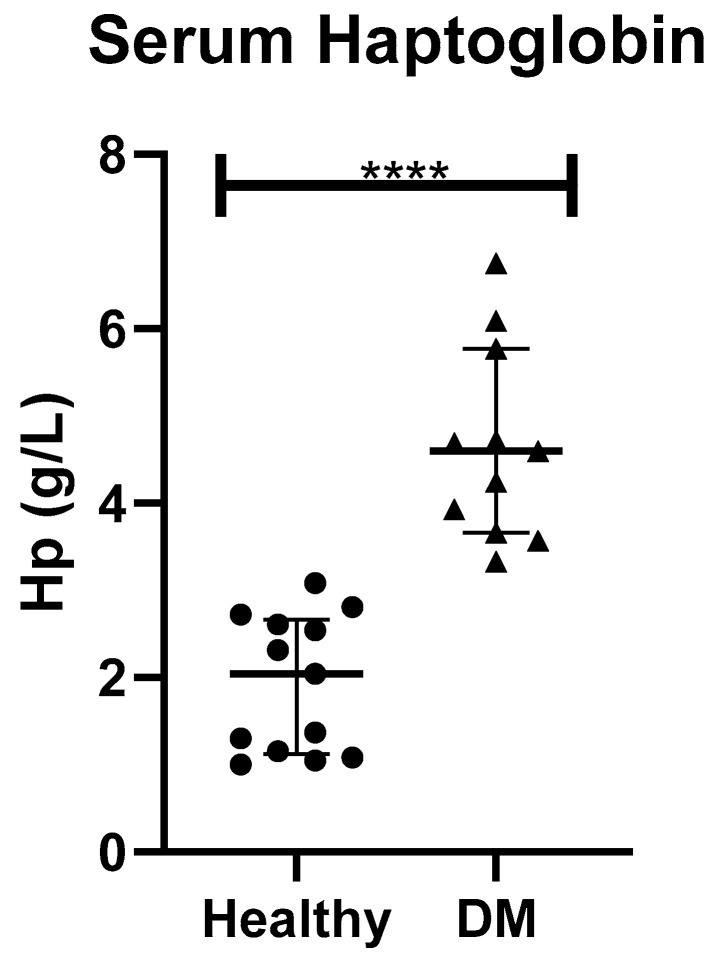
Serum haptoglobin in healthy dogs (Healthy) and dogs with type-1 diabetes mellitus (DM). Asterisks indicate differences of statistical relevance between the two groups (****: *p* > 0.001).

When haptoglobin (Hp) was measured in serum, it was statistically significantly higher in dogs with DM (median (25–75th percentile)), (4.6 (3.66–5.77) g/L) than in healthy controls (2.04 (1.12–2.67) g/L) (*p* < 0.001), as shown in Figure 2.

In the case of S100A12 verification in saliva of dogs, most samples from HC and DM showed absorbance below the Blank standard.

## 3. Discussion

The present study demonstrates, for the first time, changes occurring in saliva and serum proteomes in dogs with diabetes mellitus. Almost twice proteins were identified in saliva compared to serum, which was in concordance with other studies [22]. None of the proteins differentially modulated in DM appeared in both serum and saliva and data about molecular function, biological process, pathways or protein class was different between the two biofluids. These findings were consistent with previous works in which matched saliva and serum samples were used [23]. Therefore, both biofluids present complementary information and may have diagnostic potential for DM.

In serum, 16 proteins were found to be deregulated in DM in comparison to HC. According to PANTHER classification system, catalytic activity was the most represented molecular function. Similarly, metabolic process was the biological process most represented, including proteins such as transferrin, haptoglobin and apolipoprotein D. Transferrin is a negative acute-phase protein in dogs [24] and has anti-oxidant potential. DM is associated with mild inflammation [25], which may explain the down-regulation of transferrin observed in dogs with DM in the present study. However, there is a controversy on the status of transferrin in human diabetes, in which decreased, normal and increased levels have been reported [26,27,28]. To the best of the authors’ knowledge, this is the first report describing transferrin concentrations in the serum of dogs with DM. Haptoglobin is a positive moderate acute phase protein in dogs, increasing in serum for 2-5-fold in inflammation and also has antioxidant capacity by binding the oxidant free hemoglobin [29]. Hp was selected for verification because was the most increased protein in serum of dogs with DM in comparison to HC and commercial methods are available for its measurement. In agreement with our results, Hp in serum has been proposed as a biomarker of virus-induced type 1 DM as well as for autoimmune DM in rats [30]. Moreover, Hp gene has been associated with the increasing of DM complications in humans including nephropathy, cardiovascular diseases and retinopathies, as reviewed previously [31]. Our study showed lower apolipoprotein D (APOD) in serum of dogs with DM. Dogs with DM commonly have hypercholesterolemia with increased HDL [7] and a higher risk of atherosclerosis compared to healthy dogs [32]. In dogs, increases in apolipoproteins such as apolipoprotein E [33] and B [34] were reported in DM; however, to the best of the authors’ knowledge, this is the first time in which APOD is related to DM in dogs. Thus, since various functions in several metabolic processes have been attributed to APOD [35], further studies are desirable to clarify its role in relation to canine DM.

A total of 26 proteins showed different abundance in saliva between HC and DM groups. According to PANTHER, the majority of differentially regulated proteins in saliva are associated with catalytic activity and binding molecular functions.

Heat shock cognate 71 kDa protein (HSPA8), also known as Hsc70 or Hsp73, was the most dysregulated protein among those presenting catalytic activity in saliva, with a significant increase in dogs with DM. This. protein was reported to be in higher concentrations in plasma and retina of rats with DM when compared to healthy controls [36,37]. In humans, the polymorphisms of HSPA8 gene are significantly associated with the prevalence of hypertension [38], being hypertension also described in dogs with DM [39,40]. Additionally, distal renal tubular cells treated with 25 mM glucose showed higher increased levels of HSP70 in comparison to untreated ones (5 mM glucose) [41]. Furthermore, concentrations in HSPA8 levels were associated with cancers, neurodegenerative diseases and ageing, among other conditions [42,43]. Besides, other potential biomarkers having catalytic activity that were significantly altered in saliva of dogs with DM, including triosephosphate isomerase (TPI1) [44] and (ENO2) [45], were previously related to human DM. In the proteins related to binding molecular function, S100A proteins—a family of calcium-binding proteins involved in innate immune response- were among the most up-regulated in saliva of dogs with canine DM in the present study. Our results are in agreement to previous studies reporting higher levels of S100A9 in saliva of diabetic human adults [46] and children [16], in which S100A9 were proposed as a predictive factor for diabetes-related microvascular complications [46,47]. Similarly, increased plasma S100A12 has been related to diabetic retinopathy and might predict future major adverse endpoints in human diabetic patients [48].

Since S100A12 protein was the protein most up-regulated in saliva in DM in our study, we aimed to verify our results using a commercially available ELISA kit in an independent sample set. However, we were unable to validate our proteomic results since most samples were observed to be below the Blank standard. S100A2 in saliva has been proposed as a candidate biomarker for the early detection of oral squamous cell carcinomas in humans [49] and S100A11 is overexpressed in several human cancers tissues [50,51], although to the author’s knowledge there are no reports that evaluate these two proteins in DM in humans.

Calmodulins such as CALM2 and CALM3 are calcium-binding messengers implied in the control of insulin release from the beta cells [52]. Transgenic mice lines with alterations in CALM gene resulting in increased CALM levels in beta cells promoted the development of severe diabetes mellitus within hours of birth [53], which is in concordance with our results showing higher CALM in dogs with DM. In humans, calcium/calmodulin-dependent protein kinase II has been proved to be modulated by oxidation in several conditions including asthma, cardiovascular diseases, acute ischemic stroke, cancer and DM, as reviewed elsewhere [54]. Although CALM has been isolated and purified from dog’s pancreas [55], their presence in saliva and possible relationship with canine DM is described here for the first time.

The present study has some limitations. First, although the sample size was higher than the minimum of three replicates required for proteomics studies and is in accordance with other studies [56,57,58], it is relative small and a power analysis test was not performed. Therefore, this should be considered as a pilot study and our results should be further confirmed and validated in larger cohorts. In case of serum, a verification of the results obtained for one protein, the haptoglobin, was made with a spectrophotometric method in a larger number of samples; however, in saliva, the ELISA kit used for the verification of the S100A12, which was the protein that showed the larger variation, was not sensitive enough for the detection of this protein when a larger number of samples were analyzed. Ideally, more sensitive immune-assays should be developed for the measurement of this analyte in saliva samples. Another limitation is that dogs of different breeds, body mass index and sex were used in our study, being further studies needed to evaluate their possible influence in DM proteomes [59,60,61,62].

## 4. Methods

Ethics approval: All the procedures were written-approved by the Ethics Committees of the University of Murcia and Ministry of agriculture, livestock, fishing and aquaculture, Region of Murcia (A13170503). Written informed consents were obtained from the animals’ owners. Availability of data and materials: The datasets used and analyzed during the current study are available from the corresponding author on reasonable request.

### 4.1. Animals

A total of 10 client-owned dogs presented to private veterinary clinics of Murcia Region, Spain, during 2019 were involved in this study. Five animals (3 females; 3 mixed breeds, 1 Pinscher and 1 German shepherd; aged 8.6 ± 3.6 years) were classified as healthy based on hematology, biochemistry and a complete physical examination and included as the healthy control group (HC). Five animals (4 females; 4 mixed breeds and 1 Yorkshire Terrier; 9.6 ± 1.98 years old) were diagnosed with diabetes mellitus based on clinical signs including polyuria, polydipsia and polyphagia and laboratory findings including glycosuria and hyperglycemia (glucose > 200 mg/dL) as previously described [63] and therefore included in diabetes mellitus (DM) group (Table 3). All DM dogs responded to insulin treatment, being suggestive of insulin-dependent DM (type 1-DM). In all cases, dogs were excluded if they were suspected to be affected by concurrent diseases based on physical examination and laboratory analyses and if gingivitis was detected.

### 4.2. Saliva and Serum Sampling

Animals did not eat or exercise for at least two hours prior to their participation in the study. Saliva samples were obtained as previously reported [64] immediately before blood collection for routine clinical biochemical analyses. Saliva was collected using a small sponge placed in the mouth of the dog until it was thoroughly moistened. The sponge was then tucked into collection devices (Salivette saliva collection tube/V-Bottom, Sarstedt, Aktiengesellschaft & Co, Nümbrecht, Germany) and centrifuged (3000× *g*) for 10 min, 4 °C. Finally, the supernatants were passed to plastic tubes (Eppendorf, Hamburg, Germany) and stored at −80 °C until analysis.

Surplus serum samples, remaining after routine clinical biochemical analyses, were used for the present study. Whole blood was obtained by venipuncture of the jugular or cephalic vein and stored in tubes containing a coagulation activator and a gel separator until visible clot reaction. Samples were centrifuged (3500× *g*, 10 min) and the supernatant was stored −80 °C until analysis.

### 4.3. Proteomics Study of Saliva and Serum Samples and LC-MS/MS Analysis

For each sample, thirty-five µg of proteins were subjected to reduction, alkylation, digestion and labelling using 6-plex Tandem Mass Tag reagents, according to manufacturer instructions (Thermo Scientific, New York, NY, USA) as described previously [18,19]. A pool with 35 µg protein from each sample was included as internal standard and data from each sample and protein was calculated as a ratio of the internal standard.

The liquid chromatography tandem mass spectrometry (LC-MS/MS) analysis was performed using Dionex Ultimate 3000 RSLC nano-flow system (Dionex, Camberley, UK) and Orbitrap Q Exactive Plus mass spectrometer (Thermo Fisher Scientific, Waltham, MA, USA) as described elsewhere [58]. For peptide identification and relative quantification, SEQUEST algorithm, Proteome Discoverer (version 2.0., Thermo Fisher Scientific), was used. NCBI database search against *Canis Lupus* FASTA files was performed considering two trypsin missed cleavage sites, precursor tolerance of 10 ppm and fragment mass tolerance of 0.02 Da. The false discovery rate (FDR) for peptide identification was set at 1% and Percolator algorithm within the Proteome Discoverer workflow was used.

### 4.4. Statistical Analysis

Proteins with less than two unique peptides and outliers were removed from the analysis. Sample outliers were detected per each group and protein using Dixon’s test from R package outliers v0.14 [65]. If any sample outlier was significant (*p* < 0.05) it was removed from further analysis. Then, proteins that were missing data in >2 samples per group were removed. To test the difference in protein abundance between groups Wilcoxon-Mann-Whitney test was performed, since the majority of the analyzed proteins did not follow normal distribution, tested by Shapiro-Wilk test. Fold change between two groups was calculated as mean DM group divided by mean HC group. All statistics were performed using R v3.2.2 [66].

PCA and heatmaps were designed using R packages ggplot2 v3.1.1 [67] and pheatmap v1.0.12 [68], respectively.

Proteins GI accession numbers were converted into official gene symbol either by UniProtKB ID mapping (https://www.uniprot.org/uploadlists/), DAVID conversion tool (https://david.ncifcrf.gov/conversion.jsp) or from SEQUEST search engine implemented into Proteome Discoverer [21,69]. Genes encoding the differentially abundant proteins between DM and HC groups were used to determine the GO terms over-represented in DM using Protein Analysis Through Evolutionary Relationships (PANTHER) classification tool (http://www.pantherdb.org/).

For the validation of haptoglobin in serum, distribution of data was evaluated using D’Agostino & Pearson omnibus normality test. Since the data were not normally distributed, the non-parametric statistical Mann Whitney U (two-way) test was used to compare serum haptoglobin between the different groups.

### 4.5. Validation of Proteomic Results

Haptoglobin (Hp) concentrations in serum were determined by use of the spectrophotometric hemoglobin-binding method with the use of a commercial kit (Tridelta Development Ltd., Kildare, Ireland), following manufacturer’s instructions. The method was previously validated for its use in canine samples [70] and an automatic analyzer (AU 600 automated biochemical analyzer, Olympus, Minneapolis, MI, USA) was employed for Hp measurement. Hp was expressed in g/L.

For the verification of S100A12 protein in saliva, a commercially available ELISA Kit for S100A2 Calcium Binding Protein A12 (S100A12) (SEB080Hu, Cloud Clone, TX, USA) following manufacturer’s instructions.

For the validation of Hp and S100A12, serum and saliva samples, respectively, from 13 healthy (6 different breeds) and 11 dogs diagnosed with DM (4 different breeds) were employed.

## 5. Conclusions

In conclusion, the data from the present study highlights the potential of the TMT-based approach for the screening of changes in serum and saliva proteome in dogs with diabetes mellitus. Serum proteome analysis pointed out alterations in oxidative status, defense and inflammation proteins, being some of them such as transferrin and Hp not described previously in canine DM. Saliva proteome evidenced changes in cellular and metabolic processes related to the pathogenesis of DM, insulin resistance and possible relationship with the increased susceptibility to secondary diseases due to DM. Some of the modulated proteins in saliva such as HSPA8 or S100A9 have been previously related to DM complications such as hypertension or micro-vascular alterations, while others such as S100A2 and S100A11 are described in DM here for the first time.

Based on these results, from the clinical point of view, the proteins identified here are a useful tool to improve the knowledge about the pathogenesis of DM and may have potential use as biomarkers of this disease.

## Figures and Tables

**Table 1:** Proteins in serum with significantly differential abundances between healthy controls (HC) and dogs with diabetes mellitus (DM) identified using a Tandem Mass Tags (TMT)-based proteomic analysis.

Gene Symbol	Description	*p*-Value	Fold Change	Mean (SD) Healthy	Mean (SD) Diseased
Proteins down-regulated in DM when compared to HC					
IL13RA2	Interleukin-13 receptor subunit alpha-2 precursor [Canis lupus familiaris]	0.016	0.426	0.941 (1.062)	0.569 (0.032)
IGHG	Immunoglobulin gamma heavy chain D [*Canis lupus familiaris*]	0.016	0.426	0.941 (1.062)	0.569 (0.032)
IGLL	Immunoglobulin lambda-1 light chain [*Canis lupus familiaris*]	0.016	0.519	1.235 (0.037)	0.754 (0.173)
VPREB1	Immunoglobulin iota chain-like [*Canis lupus familiaris*]	0.016	0.538	1.179 (0.06)	0.673 (0.138)
TFRC	Transferrin receptor [*Canis lupus familiaris*]	0.016	0.664	1.149 (0.048)	0.715 (0.2)
GSN	Gelsolin [*Canis lupus familiaris*]	0.032	0.671	1.191 (0.117)	0.795 (0.103)
IGHV	Immunoglobulin heavy chain variable region [*Canis lupus familiaris*]	0.032	0.718	1.029 (0.085)	0.884 (0.315)
ITIH2	Inter-alpha-trypsin inhibitor heavy chain H2 [*Canis lupus familiaris*]	0.008	0.735	1.076 (0.112)	0.916 (0.15)
ITIH4	Inter-alpha-trypsin inhibitor heavy chain H4 [*Canis lupus familiaris*]	0.032	0.796	0.876 (0.071)	0.704 (0.056)
ITIH1	Inter-alpha-trypsin inhibitor heavy chain H1 [*Canis lupus familiaris*]	0.032	0.809	1.124 (0.043)	0.881 (0.064)
A2M	Alpha-2-macroglobulin [*Canis lupus familiaris*]	0.008	0.813	1.031 (0.076)	0.896 (0.157)
APOD	Apolipoprotein D [*Canis lupus familiaris*]	0.032	0.841	0.999 (0.144)	0.86 (0.072)
PLG	Plasminogen [*Canis lupus familiaris*]	0.032	0.883	1.09 (0.071)	0.944 (0.034)
Proteins up-regulated in DM when compared to HC					
C4BPB	C4b-binding protein beta chain [*Canis lupus dingo*]	0.032	1.341	1.03 (0.126)	1.343 (0.146)
MGAM	Maltase-glucoamylase, intestinal [*Canis lupus familiaris*]	0.016	1.367	0.83 (0.026)	1.061 (0.242)
HP	Haptoglobin heavy chain [dogs, Peptide, 245 aa]	0.032	1.584	0.537 (0.15)	0.972 (0.21)

IQR: interquartile range.

**Table 2:** Proteins in saliva with significantly differential abundances between healthy controls (HC) and dogs with diabetes mellitus (DM) identified using TMT-based proteomic analysis.

Gene Symbol	Description	*p*-Value	Fold Change	Mean (IQR) Healthy	Mean (IQR) Diseased
Proteins down-regulated in DM when compared to HC					
A1BG	Alpha-1B-glycoprotein [*Canis lupus familiaris*]	0.016	0.438	0.932 (0.541)	0.498 (0.038)
KLK1	Kallikrein-1-like [*Canis lupus dingo*]	0.029	0.452	1.599 (0.094)	0.753 (0.194)
ENO2	Gamma-enolase [*Canis lupus dingo*]	0.032	0.469	1.661 (1.274)	1.121 (0.335)
CANF2	Precursor Can f II [*Canis lupus familiaris*]	0.031	0.47	2.459 (0.632)	0.981 (0.367)
GAPDH	Glyceraldehyde-3-phosphate dehydrogenase [*Canis lupus familiaris*]	0.016	0.667	1.058 (0.467)	0.824 (0.004)
Proteins up-regulated in DM when compared to HC					
MDH1	Malate dehydrogenase, cytoplasmic [*Canis lupus familiaris*]	0.016	1.313	0.657 (0.118)	0.91 (0.123)
IPSG	Double-headed protease inhibitor, submandibular gland [*Canis lupus familiaris*]	0.029	1.39	0.698 (0.092)	0.854 (0.206)
TPI1	Triosephosphate isomerase	0.032	1.445	0.851 (0.194)	0.985 (0.113)
TKT	Transketolase [*Canis lupus familiaris*]	0.032	1.463	0.648 (0.397)	1.061 (0.086)
CALM3	Calmodulin-3 [*Canis lupus dingo*]	0.016	1.584	0.788 (0.199)	1.088 (0.054)
NME2	Nucleoside diphosphate kinase B [*Canis lupus familiaris*]	0.016	1.624	0.547 (0.248)	1.052 (0.139)
HSPA8	Heat shock cognate 71 kDa protein [*Canis lupus familiaris*]	0.032	1.666	0.585 (0.39)	1.081 (0.175)
YWHAE	14-3-3 protein epsilon [*Canis lupus familiaris*]	0.032	1.671	0.672 (0.254)	1.135 (0.17)
LCP1	Plastin-2 [*Canis lupus familiaris*]	0.016	1.684	0.538 (0.622)	1.111 (0.161)
YWHAQ	14-3-3 protein theta [*Canis lupus dingo*]	0.032	1.702	0.785 (0.286)	1.191 (0.193)
CALM2	Calmodulin-2 [*Canis lupus dingo*]	0.008	1.822	0.88 (0.298)	1.341 (0.488)
S100A2	Protein S100-A2 [*Canis lupus dingo*]	0.016	1.840	0.541 (0.151)	0.962 (0.03)
MDH2	Malate dehydrogenase, mitochondrial-like [*Canis lupus familiaris*]	0.008	1.969	0.447 (0.139)	0.88 (0.281)
CANF1	Major allergen Can f 1	0.016	1.978	0.492 (0.041)	0.88 (0.461)
FLNA	Filamin-A [*Canis lupus familiaris*]	0.032	2.102	0.464 (0.437)	1.163 (0.117)
GSTA4	Glutathione S-transferase A4-like [*Canis lupus familiaris*]	0.032	2.105	0.43 (0.19)	1.082 (0.258)
PTMA	Prothymosin alpha [*Canis lupus familiaris*]	0.032	2.348	0.432 (0.155)	1.056 (0.286)
S100A11	Protein S100-A11 [*Canis lupus familiaris*]	0.032	2.451	0.396 (0.246)	1.136 (0.528)
ARPC4	Actin-related protein 2/3 complex subunit 4 [*Canis lupus familiaris*]	0.016	2.723	0.246 (0.384)	1.161 (0.118)
S100A9	Protein S100-A9 [*Canis lupus familiaris*]	0.032	2.856	0.254 (0.442)	1.327 (0.417)
S100A12	Protein S100-A12-like [*Canis lupus dingo*]	0.032	2.963	0.268 (0.526)	1.199 (0.45)

IQR: interquartile range.

**Table 3:** Clinical history and main exploratory findings in dogs with diabetes mellitus (DM) and healthy controls (H).

ID	Sex	Age (y)	Breed	Body Weight (kg)/Diet	Glucose (mg/dL)	Fructosamine (µmol/L)	Clinical Signs
DM1	Female	12	Mixed breed	9.5/Dry food	320	387	Diabetic Retinopathy PU-PD-PF
DM2	Female (neutered)	10	Mixed breed	14.5/Dry food	337	561	Diabetic Retinopathy PU-PD-PF
DM3	Male (neutered)	6.5	Mixed breed	6/Dry food	582	372	PU-PD-PF
DM4	Male	10	Mixed breed	20/Dry food	277	497	PU-PD-PF
DM5	Female	6.5	Yorkshire Terrier	3/Dry food	212	401	PU-PD-PF
H1	Male	7	Poodle	5.7/mixed	103	267	-
H2	Male (neutered)	13	Mixed breed	14/Dry food	98	181	-
H3	Female	6	German Shepherd	28/Dry food	78	239	-
H4	Female	2	Mixed breed	15/Dry food	82	152	-
H5	Female (neutered)	5	Mixed breed	4/Dry food	106	226	-

PU-PD-PF: polyuria, polydipsia and polyphagia. Reference ranges: 70–110 mg/dL for glucose and 162–310 µmol/L for fructosamine.

## Supplementary Materials

The following are available online at https://www.mdpi.com/2076-2615/10/12/2261/s1, Figure S1. Principal component analysis (PCA) results from serum samples. Figure S2. Heatmap showing the relative abundance (color) of serum proteins in healthy (HC) and dogs with diabetes mellitus (DM). Figure S3. Principal component analysis (PCA) results from saliva samples. Figure S4. Heatmap showing the relative abundance (color) of saliva proteins in healthy (HC) and dogs with diabetes mellitus (DM). Table S1. MS/MS spectra info, peptide spectrum matches (PSMs), identified peptides, proteins and protein groups in serum samples. Table S2. MS/MS spectra info, peptide spectrum matches (PSMs), identified peptides, proteins and protein groups in saliva samples. Table S3. Serum proteins identified in dogs with diabetes mellitus (DM) and healthy controls (HC).

## Abbreviations

ApoD: apolipoprotein D; CALM: calmodulin; DM: diabetes mellitus; ENO2: enolase 2; FDR: false discovery rate; HC: healthy controls; Hp: haptoglobin; HSPA8: heat shock cognate 71 kDa protein; PANTHER: Protein Analysis Through Evolutionary Relationships; TMT: Tandem Mass Tags; TPI: triosephosphate isomerase.
