# Supplementary material for: The Serum and Saliva Proteome of Dogs with Diabetes Mellitus
Source: Animals (Basel). 2020 Dec 1;10(12):2261. doi: 10.3390/ani10122261 (PMC7760505; doi:10.3390/ani10122261)
Supplement: Supplementary file 1 [file animals-10-02261-s001.zip › animals-996011-SI.pdf]

animals

Article

The Serum and Saliva Proteome of Dogs with Diabetes Mellitus

Lorena Franco-Martínez, Andrea Gelemanović, Anita Horvatić, María Dolores Contreras-Aguilar, Vladimir Mrljak, José J. Cerón, Silvia Martínez-Subiela and Asta Tvarijonaviciute

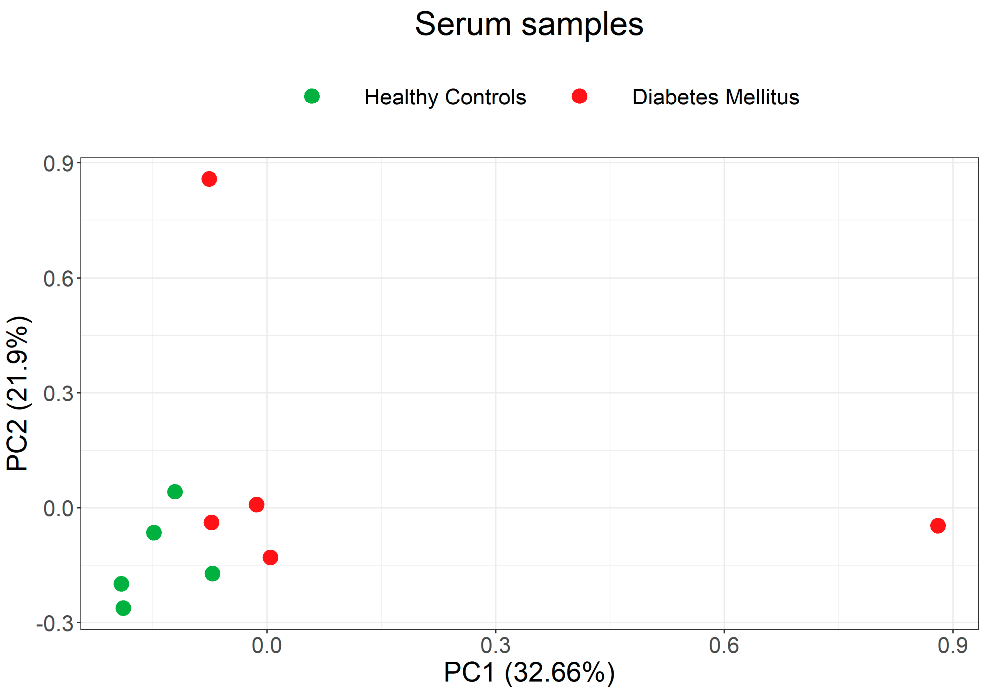

Figure S1. Principal component analysis (PCA) results from serum samples.

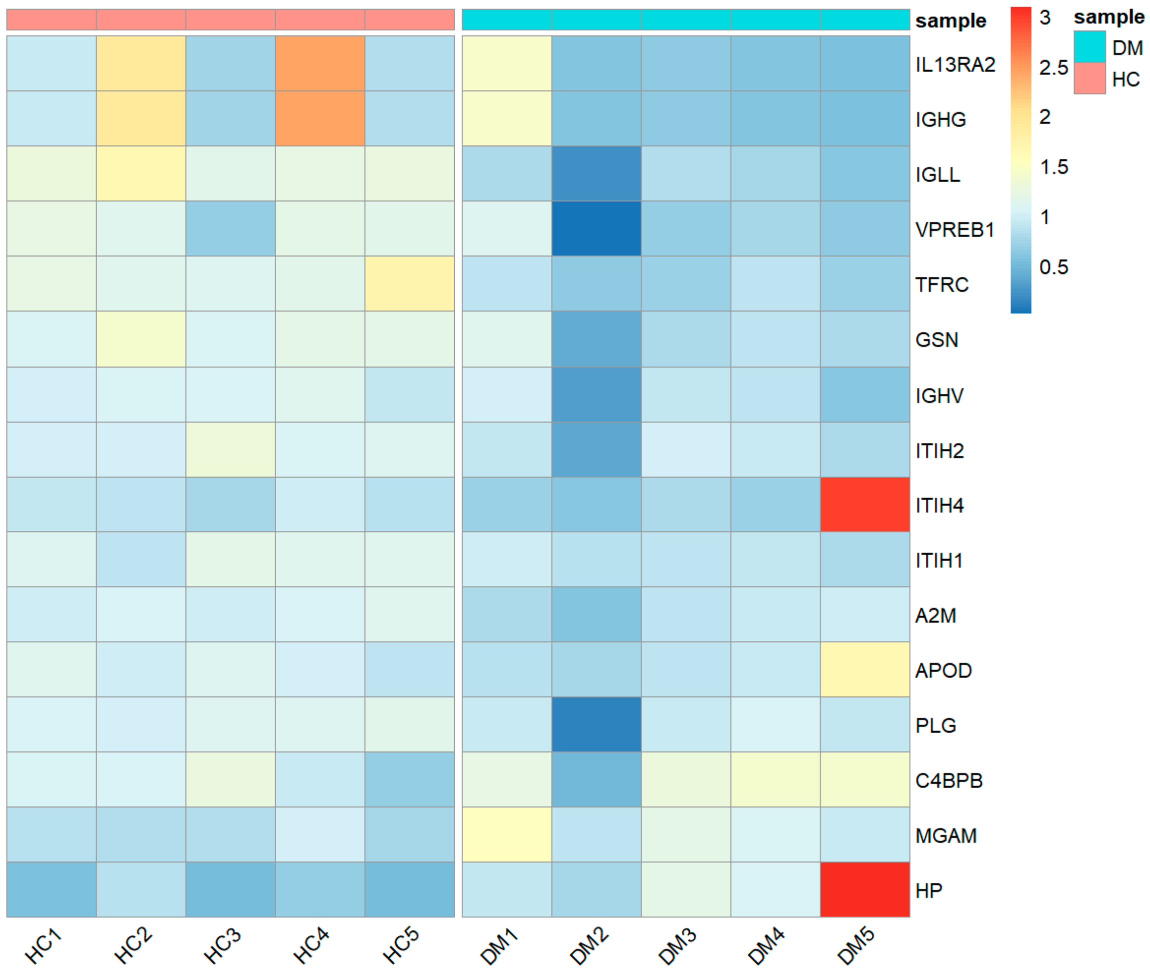

Figure S2. Heatmap showing the relative abundance (color) of serum proteins in healthy (HC) and dogs with diabetes mellitus (DM).

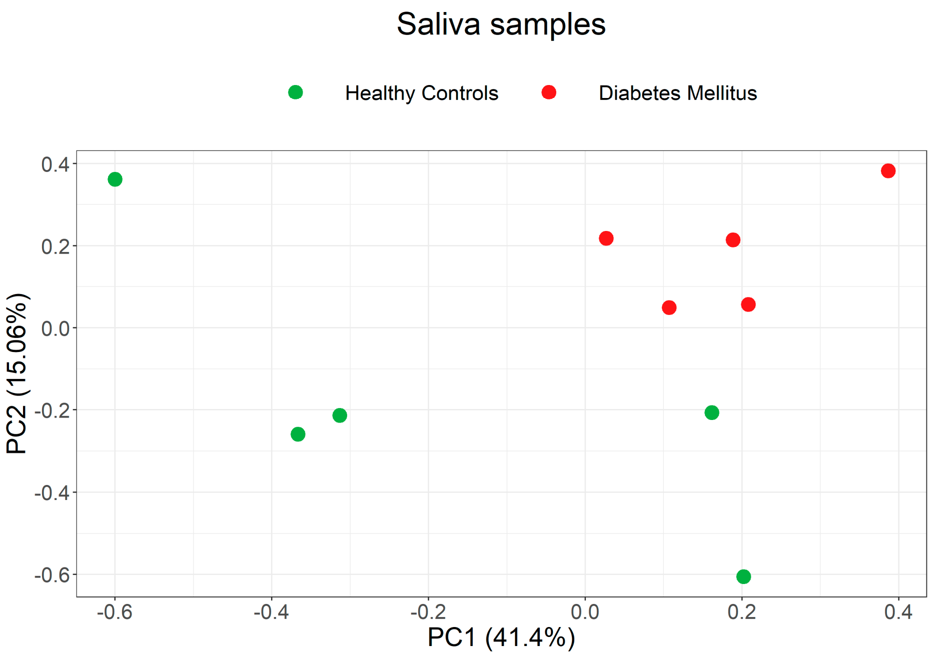

Figure S3. Principal component analysis (PCA) results from saliva samples.

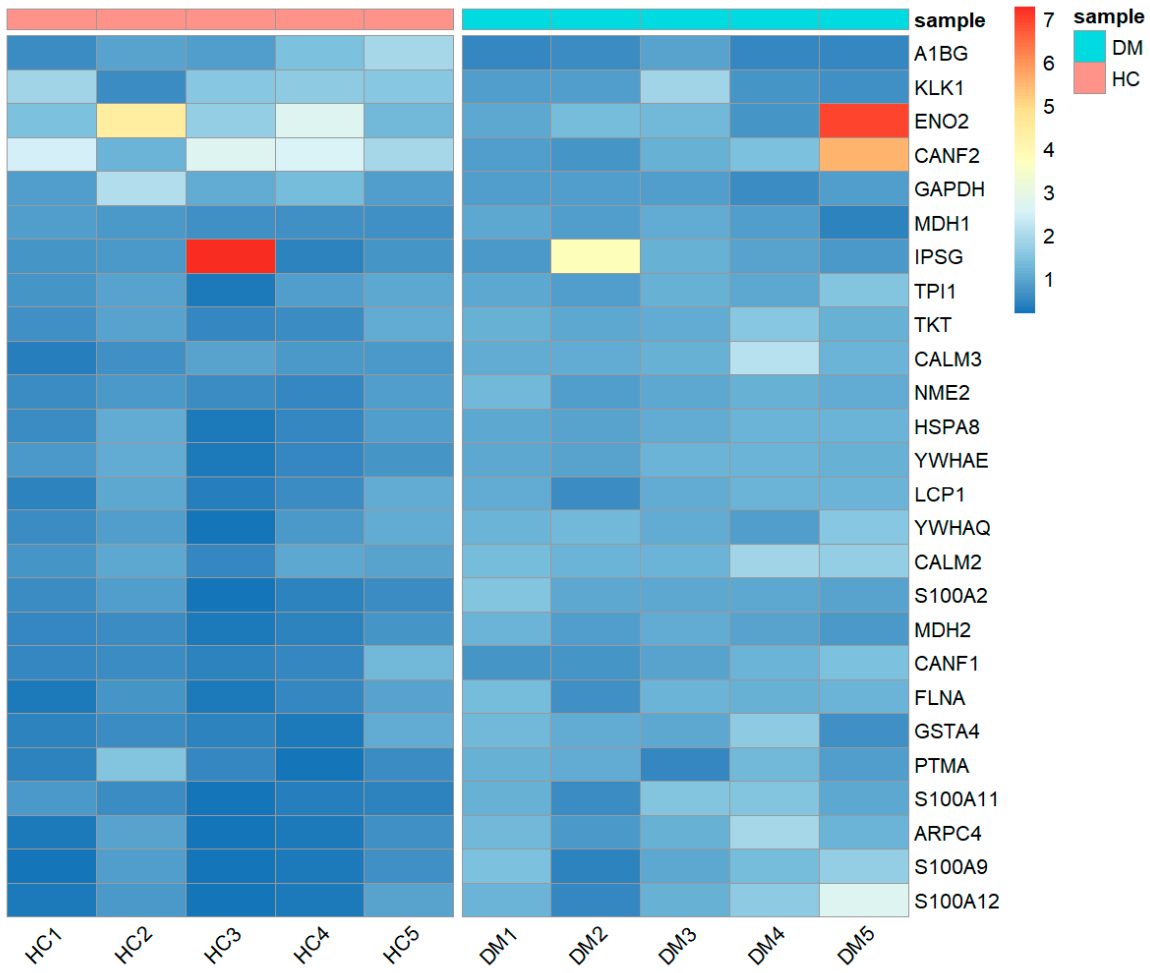

Figure S4. Heatmap showing the relative abundance (color) of saliva proteins in healthy (HC) and dogs with diabetes mellitus (DM).

Table S3. Serum proteins identified in dogs with diabetes mellitus (DM) and healthy controls (HC).

Accession Number	No. Unique Peptides	Protein Function	Median (IQR) Healthy	Median (IQR) DM	*p*
123511	30	RecName: Full=Haptoglobin; Contains: RecName: Full=Haptoglobin alpha chain; Contains: RecName: Full=Haptoglobin beta chain	0.573 (0.139)	0.995 (0.303)	0.111
130314	16	RecName: Full=Plasminogen; Contains: RecName: Full=Plasmin heavy chain A; Contains: RecName: Full=Plasmin light chain B	1.042 (0.119)	0.923 (0.097)	0.095
163903	3	apolipoprotein C-II precursor [Canis lupus familiaris]	1.044 (0.18)	1.5 (0.401)	0.310
163905	2	apolipoprotein C-III precursor [Canis lupus familiaris]	0.942 (0.197)	1.244 (1.019)	0.310
163948	3	factor IX [Canis lupus familiaris]	0.856 (0.016)	0.994 (0.198)	0.400
163954	14	glycoprotein 80 [Canis lupus familiaris]	0.907 (0.044)	0.879 (0.063)	0.841
164060	7	serum amyloid A protein, partial [Canis lupus familiaris]	0.15 (0.57)	0.117 (0.176)	0.786
164064	7	serum amyloid A protein, partial [Canis lupus familiaris]	0.15 (0.57)	0.117 (0.176)	0.786
164066	7	serum amyloid A protein, partial [Canis lupus familiaris]	0.15 (0.57)	0.117 (0.176)	0.786

164068	7	serum amyloid A protein, partial [Canis lupus familiaris]	0.15 (0.57)	0.117 (0.176)	0.786
227017	3	amyloid A protein	0.062 (0.457)	0.046 (0.128)	0.460
258498	4	haptoglobin light chain, HpL chain [dogs, Peptide, 83 aa]	0.806 (0.139)	1.514 (0.914)	0.421
258499	25	haptoglobin heavy chain, HpH chain [dogs, Peptide, 245 aa]	0.537 (0.15)	0.972 (0.21)	0.032
296089	13	apolipoprotein H; beta-2-glycoprotein I [Canis lupus familiaris]	0.989 (0.047)	1.123 (0.276)	0.310
598107	9	IgA heavy chain constant region, partial [Canis lupus familiaris]	0.577 (0.083)	1.464 (0.878)	0.151
1096665	2	Ig:SUBUNIT=epsilon	1.646 (0.238)	0.758 (0.082)	0.333
1222669	2	beta-actin, partial [Canis lupus familiaris]	0.963 (0.086)	0.701 (0.408)	0.100
1272412	8	fibronectin, partial [Canis lupus familiaris]	1.166 (0.107)	1.006 (0.187)	0.556
1272414	11	fibronectin, partial [Canis lupus familiaris]	1.152 (0.029)	0.956 (0.323)	0.190
1304047	12	fibrinogen A-alpha-chain, partial [Canis lupus familiaris]	0.413 (0.074)	0.449 (0.107)	0.548
2147092	2	albumin - dog (fragment)	1.068 (0.099)	1.053 (0.069)	0.548
3319897	2	albumin [Canis lupus familiaris]	1.067 (0.071)	1.053 (0.022)	0.095
3328199	4	coagulation factor X, partial [Canis lupus familiaris]	1.038 (0.042)	1.014 (0.122)	0.629
3915605	11	RecName: Full=Apolipoprotein E; Short=Apo-E	0.835 (0.113)	0.876 (0.144)	0.730
5597005	3	beta-actin [Canis lupus familiaris]	0.878 (0.057)	0.865 (0.192)	0.690
5821257	3	angiotensinogen, partial [Canis lupus familiaris]	1.077 (0.136)	1.211 (0.334)	1.000
6687188	4	albumin [Canis lupus familiaris]	0.551 (0.979)	1.311 (0.301)	0.310
10946310	8	transferrin receptor [Canis lupus familiaris]	1.149 (0.048)	0.715 (0.2)	0.016
15825495	3	adiponectin, partial [Canis lupus familiaris]	1.212 (0.103)	0.585 (0.081)	0.151
16607648	6	unnamed protein product [Canis lupus familiaris]	1.17 (1.041)	0.453 (0.321)	0.056
16607678	3	unnamed protein product, partial [Canis lupus familiaris]	0.875 (1.023)	0.636 (0.136)	0.063
16607681	8	unnamed protein product, partial [Canis lupus familiaris]	0.941 (1.062)	0.569 (0.032)	0.016
16607715	6	unnamed protein product [Canis lupus familiaris]	1.17 (1.033)	0.453 (0.32)	0.056
16607724	8	unnamed protein product [Canis lupus familiaris]	0.941 (1.062)	0.569 (0.032)	0.016
17066530	8	immunoglobulin gamma heavy chain D [Canis lupus familiaris]	0.941 (1.062)	0.569 (0.032)	0.016
18139619	13	plasminogen, partial [Canis lupus familiaris]	1.09 (0.071)	0.944 (0.034)	0.032
19715661	2	immunoglobulin J chain, partial [Canis lupus familiaris]	0.812 (0.597)	1.592 (0.373)	0.222
45826457	5	ceruloplasmin, partial [Canis lupus familiaris]	0.775 (0.161)	0.922 (0.477)	1.000
54792748	3	adiponectin precursor [Canis lupus familiaris]	1.212 (0.103)	0.585 (0.081)	0.151
55741727	3	serum amyloid A1 precursor [Canis lupus familiaris]	0.062 (0.457)	0.046 (0.128)	0.460

57036446	10	apolipoprotein E [Canis lupus familiaris]	0.828 (0.111)	0.87 (0.145)	0.413
57091057	2	membrane primary amine oxidase isoform X1 [Canis lupus familiaris]	0.89 (0.088)	1.036 (0.146)	0.063
57094349	3	apolipoprotein M isoform X1 [Canis lupus familiaris]	0.936 (0.165)	0.878 (0.115)	0.841
57102730	3	serum amyloid A protein-like [Canis lupus familiaris]	0.062 (0.457)	0.046 (0.128)	0.460
57108093	3	actin, alpha cardiac muscle 1 [Canis lupus familiaris]	0.878 (0.057)	0.865 (0.192)	0.690
60734606	2	unnamed protein product, partial [Canis lupus familiaris]	0.466 (0.075)	1.513 (0.988)	0.151
60734607	5	unnamed protein product, partial [Canis lupus familiaris]	0.694 (0.129)	1.351 (0.718)	0.310
62421376	2	vitronectin, partial [Canis mesomelas]	1 (0.022)	1.043 (0.419)	0.841
62870505	2	apolipoprotein B, partial [Canis lupus]	0.917 (0.081)	1.143 (0.266)	0.667
70909945	5	zinc alpha-2-glycoprotein 1, partial [Canis lupus familiaris]	0.98 (0.046)	1.133 (0.224)	0.400
73532760	2	hepatocyte growth factor activator precursor [Canis lupus familiaris]	0.823 (0.031)	0.874 (0)	0.667
73948526	2	xaa-Pro dipeptidase isoform X1 [Canis lupus familiaris]	1.135 (0.047)	0.967 (0.072)	0.057
73949158	18	inter-alpha-trypsin inhibitor heavy chain H2 [Canis lupus familiaris]	1.076 (0.112)	0.916 (0.15)	0.008
73953733	2	mannose-binding protein C [Canis lupus familiaris]	1.224 (0.284)	0.982 (0.496)	0.629
73953824	17	complement component C7 isoform X1 [Canis lupus familiaris]	0.95 (0.154)	0.836 (0.034)	0.095
73953994	3	coagulation factor XII isoform X1 [Canis lupus familiaris]	1.109 (0.141)	0.996 (0.159)	0.905
73956394	3	complement component C8 beta chain [Canis lupus familiaris]	1.012 (0.129)	1.06 (0.223)	1.000
73957095	30	haptoglobin-like [Canis lupus familiaris]	0.573 (0.139)	0.995 (0.303)	0.111
73964432	12	alpha-1-antichymotrypsin [Canis lupus familiaris]	0.782 (0.047)	0.854 (0.193)	0.413
73967363	5	alpha-2-antiplasmin isoform X1 [Canis lupus familiaris]	1 (0.181)	1.023 (0.12)	0.905
73967778	4	ficolin-2 isoform X1 [Canis lupus familiaris]	0.776 (0.229)	0.891 (0.302)	0.802
73975389	27	afamin [Canis lupus familiaris]	1.07 (0.125)	0.936 (0.272)	0.056
73975797	13	serum paraoxonase/arylesterase 1 [Canis lupus familiaris]	0.881 (0.14)	1.1 (0.385)	0.056
73977992	19	fibrinogen gamma chain isoform X2 [Canis lupus familiaris]	0.46 (0.024)	0.567 (0.139)	0.222
73978329	24	fibrinogen alpha chain [Canis lupus familiaris]	0.431 (0.057)	0.44 (0.096)	0.421
73985485	21	inter-alpha-trypsin inhibitor heavy chain H1 isoform X1 [Canis lupus familiaris]	1.124 (0.043)	0.881 (0.064)	0.032
73988725	15	hemopexin [Canis lupus familiaris]	0.789 (0.265)	0.877 (0.04)	0.730
73990367	25	ceruloplasmin isoform X3 [Canis lupus familiaris]	0.893 (0.116)	0.864 (0.072)	1.000
73997275	6	complement C1s subcomponent [Canis lupus familiaris]	1.017 (0.064)	1.028 (0.089)	0.556
74003556	3	fetuin-B [Canis lupus familiaris]	0.525 (0.435)	1.198 (0.586)	0.056
74007356	2	properdin [Canis lupus familiaris]	0.833 (0.329)	0.72 (0.373)	0.841

75062693	6	RecName: Full=Keratin, type II cytoskeletal 1; AltName: Full=Cytokeratin-1; Short=CK-1; AltName: Full=Epithelial keratin-1; AltName: Full=Keratin-1; Short=K1; AltName: Full=Type-II keratin Kb1	1.07 (0.285)	1.309 (4.301)	0.700
78499405	4	apolipoprotein B, partial [Canis adustus]	0.912 (0.082)	0.722 (0.126)	0.127
78499407	5	apolipoprotein B, partial [Canis aureus]	0.937 (0.108)	0.914 (0.227)	0.841
78499409	4	apolipoprotein B, partial [Canis latrans]	0.912 (0.082)	0.722 (0.126)	0.127
78499413	5	apolipoprotein B, partial [Canis mesomelas]	0.917 (0.087)	0.726 (0.124)	0.063
78499415	5	apolipoprotein B, partial [Canis simensis]	0.917 (0.087)	0.726 (0.124)	0.063
78709133	8	apolipoprotein B, partial [Canis adustus]	0.854 (0.346)	0.716 (0.476)	0.849
78709135	8	apolipoprotein B, partial [Canis aureus]	0.854 (0.346)	0.716 (0.476)	0.849
78709137	7	apolipoprotein B, partial [Canis latrans]	0.854 (0.346)	0.716 (0.476)	0.849
78709141	6	apolipoprotein B, partial [Canis mesomelas]	0.847 (0.309)	0.729 (1.739)	0.690
78709143	8	apolipoprotein B, partial [Canis simensis]	0.854 (0.346)	0.716 (0.476)	0.849
103484123	3	globin, partial [Canis lupus familiaris]	0.566 (0.165)	0.79 (0.207)	0.286
119637837	5	pigment epithelium-derived factor [Canis lupus familiaris]	1.118 (0.145)	0.992 (0.084)	0.111
124390007	3	immunoglobulin heavy chain constant region CH1, partial [Canis lupus familiaris]	0.874 (0.238)	1.012 (0.315)	0.905
124390009	3	immunoglobulin heavy chain constant region CH2, partial [Canis lupus familiaris]	0.964 (0.287)	1.036 (0.19)	0.841
124390011	2	immunoglobulin heavy chain constant region CH3, partial [Canis lupus familiaris]	0.838 (0.594)	1.075 (1.54)	0.548
124390013	4	immunoglobulin heavy chain constant region CH4, partial [Canis lupus familiaris]	1.096 (0.272)	0.972 (0.183)	0.548
146743249	3	immunoglobulin mu heavy chain variable region, partial [Canis lupus familiaris]	1.03 (0.197)	0.938 (0.17)	0.690
164430480	2	immunoglobulin lambda light chain variable region, partial [Canis lupus familiaris]	1.035 (0.096)	0.892 (0.21)	0.190
164430486	2	immunoglobulin lambda light chain variable region, partial [Canis lupus familiaris]	1.105 (0.579)	0.611 (0.154)	0.151
164430516	2	immunoglobulin lambda light chain variable region, partial [Canis lupus familiaris]	1.105 (0.579)	0.611 (0.154)	0.151
194368499	2	Chain C, Hemoglobin Subunit Alpha	0.638 (0.073)	0.768 (0.18)	0.343
204638075	3	adiponectin, partial [Canis lupus]	1.212 (0.103)	0.585 (0.081)	0.151
208342048	3	immunoglobulin heavy chain variable region, partial [Canis lupus familiaris]	1.029 (0.085)	0.884 (0.315)	0.032
208342196	3	immunoglobulin heavy chain variable region, partial [Canis lupus familiaris]	1.139 (0.133)	0.913 (0.21)	0.400
227343817	14	Chain B, Crystal Structure Of Dog (Canis Familiaris) Hemoglobin	0.626 (0.14)	0.854 (0.168)	0.286
256016957	2	apolipoprotein B, partial [Canis lupus]	0.917 (0.081)	1.143 (0.266)	0.667
269307499	2	apolipoprotein B, partial [Canis lupus]	0.917 (0.081)	1.143 (0.266)	0.667
283972739	5	apolipoprotein E4, partial [Canis lupus familiaris]	0.872 (0.132)	0.783 (0.353)	0.690
283972741	5	apolipoprotein E4, partial [Canis lupus familiaris]	0.872 (0.132)	0.783 (0.353)	0.690

283972753	5	apolipoprotein E4, partial [Canis lupus familiaris]	0.872 (0.132)	0.783 (0.353)	0.690
345777712	2	protein AMBP [Canis lupus familiaris]	1.191 (0.091)	1.087 (0.169)	0.310
345778397	27	complement factor B [Canis lupus familiaris]	1.093 (0.052)	0.939 (0.129)	0.222
345779666	5	immunoglobulin J chain [Canis lupus familiaris]	0.846 (0.388)	1.258 (0.42)	0.690
345789637	6	lipopolysaccharide-binding protein [Canis lupus familiaris]	0.63 (0.222)	0.847 (2.046)	0.229
345791439	11	heparin cofactor 2 [Canis lupus familiaris]	0.862 (0.065)	0.947 (0.05)	0.310
345792424	47	alpha-2-macroglobulin [Canis lupus familiaris]	1.031 (0.076)	0.896 (0.157)	0.008
345794639	4	thrombospondin-1 [Canis lupus familiaris]	0.907 (0.101)	1.286 (0.284)	0.667
345799905	25	apolipoprotein A-IV [Canis lupus familiaris]	0.937 (0.334)	1.097 (0.219)	0.690
359319377	3	complement component C8 alpha chain isoform X1 [Canis lupus familiaris]	1.075 (0.112)	0.95 (0.32)	0.229
359320010	23	antithrombin-III [Canis lupus familiaris]	1.096 (0.099)	0.881 (0.128)	0.056
359321961	19	prothrombin [Canis lupus familiaris]	0.986 (0.107)	0.982 (0.026)	1.000
387178037	8	transferrin receptor protein 1 [Canis mesomelas]	1.149 (0.048)	0.715 (0.2)	0.016
545487024	13	alpha-1B-glycoprotein [Canis lupus familiaris]	1.09 (0.016)	0.639 (0.367)	0.151
545489037	2	xaa-Pro dipeptidase isoform X2 [Canis lupus familiaris]	1.135 (0.047)	0.967 (0.072)	0.057
545494757	11	angiotensinogen [Canis lupus familiaris]	1.008 (0.045)	1.153 (0.298)	0.222
545494777	3	actin, alpha skeletal muscle [Canis lupus familiaris]	0.878 (0.057)	0.865 (0.192)	0.690
545495643	3	coagulation factor XII isoform X2 [Canis lupus familiaris]	1.109 (0.141)	0.996 (0.159)	0.905
545496317	6	complement component C9 [Canis lupus familiaris]	0.859 (0.216)	1.012 (0.126)	0.548
545500782	4	actin, cytoplasmic 1 isoform X1 [Canis lupus familiaris]	0.878 (0.057)	0.865 (0.192)	0.690
545504208	4	coagulation factor XIII B chain [Canis lupus familiaris]	1.068 (0.23)	1.048 (0.682)	1.000
545504920	2	coagulation factor V [Canis lupus familiaris]	0.894 (0.119)	1.087 (0.289)	0.556
545508387	3	corticosteroid-binding globulin [Canis lupus familiaris]	0.92 (0.107)	1.038 (0.325)	0.690
545508405	6	plasma serine protease inhibitor [Canis lupus familiaris]	0.875 (0.448)	0.86 (0.212)	0.889
545510243	5	pigment epithelium-derived factor isoform X1 [Canis lupus familiaris]	1.118 (0.145)	0.992 (0.084)	0.111
545512145	5	alpha-2-antiplasmin isoform X2 [Canis lupus familiaris]	1 (0.181)	1.023 (0.12)	0.905
545524897	19	fibrinogen gamma chain isoform X1 [Canis lupus familiaris]	0.46 (0.024)	0.567 (0.139)	0.222
545528321	74	apolipoprotein B-100 [Canis lupus familiaris]	0.932 (0.084)	0.782 (0.148)	0.063
545533419	17	inter-alpha-trypsin inhibitor heavy chain H1 isoform X3 [Canis lupus familiaris]	1.108 (0.144)	0.888 (0.065)	0.063
545535766	7	serum amyloid A1 isoform X1 [Canis lupus familiaris]	0.15 (0.57)	0.117 (0.176)	0.786
545536976	6	serum amyloid A protein-like [Canis lupus familiaris]	0.127 (0.18)	0.117 (0.176)	0.971

545536980	7	serum amyloid A protein isoform X1 [Canis lupus familiaris]	0.15 (0.57)	0.117 (0.176)	0.786
545544683	3	immunoglobulin lambda-1 light chain isoform X34 [Canis lupus familiaris]	1.235 (0.037)	0.754 (0.173)	0.016
545546412	57	pregnancy zone protein-like isoform X1 [Canis lupus familiaris]	1.047 (0.099)	0.869 (0.224)	0.222
545546414	58	pregnancy zone protein-like isoform X2 [Canis lupus familiaris]	1.05 (0.1)	0.861 (0.22)	0.222
545547980	3	hyaluronan-binding protein 2 [Canis lupus familiaris]	1.128 (0.185)	0.926 (0.362)	0.841
545552242	10	complement factor I isoform X3 [Canis lupus familiaris]	1.065 (0.12)	0.986 (0.046)	0.286
545553264	3	carboxypeptidase N subunit 2 [Canis lupus familiaris]	1.129 (0.262)	0.954 (0.102)	0.841
545553762	12	histidine-rich glycoprotein isoform X1 [Canis lupus familiaris]	1.019 (0.563)	1.357 (0.441)	0.841
545553764	9	histidine-rich glycoprotein isoform X2 [Canis lupus familiaris]	0.907 (0.344)	1.3 (0.585)	0.690
545554529	13	phosphatidylinositol-glycan-specific phospholipase D isoform X1 [Canis lupus familiaris]	0.913 (0.096)	1.137 (0.198)	0.063
558695388	33	plasminogen precursor [Canis lupus familiaris]	1.04 (0.118)	0.946 (0.03)	0.063
560879429	8	zinc-alpha-2-glycoprotein precursor [Canis lupus familiaris]	0.949 (0.076)	1.057 (0.316)	0.548
590121823	15	apoptosis inhibitor of macrophage [Canis lupus familiaris]	1.07 (0.078)	1.071 (0.214)	1.000
597500968	5	RecName: Full=Apolipoprotein A-II; Short=Apo-AII; Short=ApoA-II; AltName: Full=Apolipoprotein A2; Contains: RecName: Full=Proapolipoprotein A-II; Short=ProapoA-II; Contains: RecName: Full=Truncated apolipoprotein A-II; AltName: Full=Apolipoprotein A-II(1-76); Flags: Precursor	1.082 (0.11)	1.119 (0.32)	1.000
701217752	8	transferrin receptor protein 1 [Canis lupus]	1.149 (0.048)	0.715 (0.2)	0.016
704000372	21	RecName: Full=Apolipoprotein A-IV; Short=Apo-AIV; Short=ApoA-IV; AltName: Full=Apolipoprotein A4; Flags: Precursor	0.932 (0.363)	1.087 (0.211)	0.690
924183513	4	actin, cytoplasmic 1 [Canis lupus familiaris]	0.878 (0.057)	0.865 (0.192)	0.690
924442847	4	actin, cytoplasmic 2 [Canis lupus familiaris]	0.878 (0.057)	0.865 (0.192)	0.690
924859480	3	apolipoprotein C-III precursor [Canis lupus familiaris]	1.006 (0.2)	1.288 (0.836)	0.151
925718015	2	apolipoprotein B-100, partial [Canis adustus]	0.917 (0.081)	1.143 (0.266)	0.667
925718021	2	apolipoprotein B-100, partial [Canis aureus]	0.917 (0.081)	1.143 (0.266)	0.667
925718027	2	apolipoprotein B-100, partial [Canis aureus]	0.917 (0.081)	1.143 (0.266)	0.667
925718029	2	apolipoprotein B-100, partial [Canis aureus]	0.917 (0.081)	1.143 (0.266)	0.667
925718033	2	apolipoprotein B-100, partial [Canis latrans]	0.917 (0.081)	1.143 (0.266)	0.667
925718037	2	apolipoprotein B-100, partial [Canis simensis]	0.917 (0.081)	1.143 (0.266)	0.667
925718053	2	apolipoprotein B-100, partial [Canis lupus]	0.917 (0.081)	1.143 (0.266)	0.667
925718059	2	apolipoprotein B-100, partial [Canis latrans]	0.917 (0.081)	1.143 (0.266)	0.667
928139154	3	LOW QUALITY PROTEIN: sulfhydryl oxidase 1, partial [Canis lupus familiaris]	0.906 (0.084)	0.763 (1.475)	1.000

928144438	6	keratin, type I cytoskeletal 10 isoform X1 [Canis lupus familiaris]	1.127 (0.091)	1.057 (0.389)	0.730
928144440	6	keratin, type I cytoskeletal 10 isoform X2 [Canis lupus familiaris]	1.127 (0.091)	1.057 (0.389)	0.730
928144442	6	keratin, type I cytoskeletal 10 isoform X3 [Canis lupus familiaris]	1.127 (0.091)	1.057 (0.389)	0.730
928156540	32	maltase-glucoamylase, intestinal isoform X2 [Canis lupus familiaris]	0.83 (0.026)	1.061 (0.242)	0.016
928162811	3	tetranectin [Canis lupus familiaris]	1.193 (0.141)	0.918 (0.318)	0.222
928164703	4	complement C3 [Canis lupus familiaris]	1 (0.336)	0.848 (0.076)	0.111
928166207	4	serum amyloid A protein [Canis lupus familiaris]	0.109 (0.411)	0.106 (0.117)	0.786
928167527	25	ceruloplasmin isoform X1 [Canis lupus familiaris]	0.893 (0.116)	0.864 (0.072)	1.000
928167632	3	serotransferrin [Canis lupus familiaris]	1.1 (0.095)	1.173 (0.171)	0.421
928175781	6	retinol-binding protein 4 [Canis lupus familiaris]	0.961 (0.362)	1.345 (0.933)	0.841
928175986	4	carboxypeptidase N catalytic chain isoform X1 [Canis lupus familiaris]	1.014 (0.048)	1.16 (0.281)	0.730
928179004	9	complement factor I isoform X4 [Canis lupus familiaris]	1.05 (0.118)	0.986 (0.042)	0.286
928180090	4	apolipoprotein D [Canis lupus familiaris]	0.999 (0.144)	0.86 (0.072)	0.032
928181234	11	phosphatidylinositol-glycan-specific phospholipase D isoform X3 [Canis lupus familiaris]	0.957 (0.168)	0.993 (0.266)	0.690
928182507	58	fibronectin isoform X4 [Canis lupus familiaris]	1.051 (0.094)	1.061 (0.225)	0.730
928182509	58	fibronectin isoform X5 [Canis lupus familiaris]	1.051 (0.094)	1.061 (0.225)	0.730
928182511	58	fibronectin isoform X7 [Canis lupus familiaris]	1.051 (0.094)	1.061 (0.225)	0.730
928182513	58	fibronectin isoform X9 [Canis lupus familiaris]	1.051 (0.094)	1.061 (0.225)	0.730
928182519	57	fibronectin isoform X11 [Canis lupus familiaris]	1.051 (0.095)	1.061 (0.225)	0.730
928182521	57	fibronectin isoform X1 [Canis lupus familiaris]	1.053 (0.092)	1.063 (0.199)	0.595
928182523	56	fibronectin isoform X10 [Canis lupus familiaris]	1.053 (0.093)	1.063 (0.199)	0.595
928186325	30	inter-alpha-trypsin inhibitor heavy chain H4 isoform X1 [Canis lupus familiaris]	0.876 (0.071)	0.704 (0.056)	0.032
928186327	30	inter-alpha-trypsin inhibitor heavy chain H4 isoform X3 [Canis lupus familiaris]	0.876 (0.071)	0.704 (0.056)	0.032
928186331	4	LOW QUALITY PROTEIN: inter-alpha-trypsin inhibitor heavy chain H3 [Canis lupus familiaris]	0.993 (0.116)	0.814 (0.142)	0.151
1101972892	9	TPA: globin A1 [Canis lupus familiaris]	0.594 (0.148)	0.845 (0.156)	0.286
1104685307	4	Chain A, Serum Albumin	0.551 (0.979)	1.311 (0.301)	0.310
1239883263	29	inter-alpha-trypsin inhibitor heavy chain H4 isoform X2 [Canis lupus familiaris]	0.876 (0.071)	0.704 (0.056)	0.032
1239893961	2	hepatocyte growth factor activator isoform X1 [Canis lupus familiaris]	0.823 (0.031)	0.874 (0)	0.667
1239898360	3	coagulation factor XII isoform X3 [Canis lupus familiaris]	1.109 (0.141)	0.996 (0.159)	0.905
1239899336	7	complement component C6 [Canis lupus familiaris]	0.928 (0.178)	1.011 (0.32)	0.151
1239899370	16	complement component C7 isoform X2 [Canis lupus familiaris]	0.947 (0.166)	0.826 (0.008)	0.151

1239901836	5	sex hormone-binding globulin isoform X2 [Canis lupus familiaris]	1.258 (0.456)	1.403 (0.876)	0.629
1239902894	3	complement component C8 alpha chain isoform X2 [Canis lupus familiaris]	1.075 (0.112)	0.95 (0.32)	0.229
1239918266	2	membrane primary amine oxidase isoform X3 [Canis lupus familiaris]	0.89 (0.088)	1.036 (0.146)	0.063
1239918269	2	membrane primary amine oxidase isoform X4 [Canis lupus familiaris]	0.89 (0.088)	1.036 (0.146)	0.063
1239920122	4	alpha-2-antiplasmin isoform X3 [Canis lupus familiaris]	1.044 (0.226)	1.036 (0.128)	0.730
1239920124	4	alpha-2-antiplasmin isoform X4 [Canis lupus familiaris]	1.044 (0.226)	1.036 (0.128)	0.730
1239920398	2	complement component C8 gamma chain isoform X1 [Canis lupus familiaris]	0.959 (0.026)	1.081 (0.235)	0.730
1239920689	4	ficolin-2 isoform X2 [Canis lupus familiaris]	0.776 (0.229)	0.891 (0.302)	0.802
1239925760	29	complement C5 [Canis lupus familiaris]	0.921 (0.205)	0.939 (0.071)	0.905
1239925762	14	gelsolin [Canis lupus familiaris]	1.191 (0.117)	0.795 (0.103)	0.032
1239928570	60	complement C4-A [Canis lupus familiaris]	1.031 (0.082)	0.936 (0.038)	0.190
1239928849	7	complement C2 isoform X1 [Canis lupus familiaris]	0.91 (0.011)	1.19 (0.385)	0.151
1239928853	7	complement C2 isoform X3 [Canis lupus familiaris]	0.91 (0.011)	1.19 (0.385)	0.151
1239938505	6	plasma kallikrein isoform X2 [Canis lupus familiaris]	1.074 (0.054)	1.083 (0.09)	0.730
1239938902	3	putative maltase-glucoamylase-like protein FLJ16351 isoform X1 [Canis lupus familiaris]	0.901 (0.285)	1.018 (0.516)	1.000
1239938904	3	putative maltase-glucoamylase-like protein FLJ16351 isoform X2 [Canis lupus familiaris]	0.901 (0.285)	1.018 (0.516)	1.000
1239938912	32	maltase-glucoamylase, intestinal isoform X1 [Canis lupus familiaris]	0.83 (0.026)	1.061 (0.242)	0.016
1239944268	7	plasma protease C1 inhibitor [Canis lupus familiaris]	0.979 (0.222)	0.802 (0.127)	0.278
1239949881	20	inter-alpha-trypsin inhibitor heavy chain H1 isoform X2 [Canis lupus familiaris]	1.124 (0.033)	0.895 (0.03)	0.056
1239957836	25	ceruloplasmin isoform X2 [Canis lupus familiaris]	0.893 (0.116)	0.864 (0.072)	1.000
1239957839	25	ceruloplasmin isoform X4 [Canis lupus familiaris]	0.893 (0.116)	0.864 (0.072)	1.000
1239964876	2	immunoglobulin lambda-1 light chain isoform X32 [Canis lupus familiaris]	1.14 (0.423)	0.762 (0.056)	0.222
1239964884	2	immunoglobulin lambda-1 light chain isoform X37 [Canis lupus familiaris]	1.14 (0.423)	0.762 (0.056)	0.222
1239964904	2	immunoglobulin lambda-1 light chain isoform X48 [Canis lupus familiaris]	1.14 (0.423)	0.762 (0.056)	0.222
1239964906	2	immunoglobulin lambda-1 light chain isoform X49 [Canis lupus familiaris]	1.14 (0.423)	0.762 (0.056)	0.222
1239965532	2	immunoglobulin iota chain-like [Canis lupus familiaris]	1.179 (0.06)	0.673 (0.138)	0.016
1239967247	50	pregnancy zone protein-like isoform X3 [Canis lupus familiaris]	1.01 (0.049)	1.007 (0.202)	0.421
1239976293	10	complement factor I isoform X1 [Canis lupus familiaris]	1.065 (0.12)	0.986 (0.046)	0.286
1239976295	10	complement factor I isoform X2 [Canis lupus familiaris]	1.065 (0.12)	0.986 (0.046)	0.286
1239976411	16	vitamin K-dependent protein S [Canis lupus familiaris]	0.96 (0.143)	1.026 (0.061)	0.310
1239977620	3	adiponectin isoform X2 [Canis lupus familiaris]	1.212 (0.103)	0.585 (0.081)	0.151

1239982239	57	fibronectin isoform X2 [Canis lupus familiaris]	1.053 (0.092)	1.063 (0.199)	0.595
1239982241	57	fibronectin isoform X3 [Canis lupus familiaris]	1.053 (0.092)	1.063 (0.199)	0.595
1239982243	56	fibronectin isoform X6 [Canis lupus familiaris]	1.053 (0.093)	1.063 (0.199)	0.595
1239982245	54	fibronectin isoform X8 [Canis lupus familiaris]	1.046 (0.098)	1.046 (0.18)	0.730
1340236572	2	immunoglobulin heavy chain variable region, partial [Canis lupus familiaris]	1.207 (0.759)	0.655 (0.056)	0.063
1418198271	3	LOW QUALITY PROTEIN: carboxypeptidase N subunit 2-like [Canis lupus dingo]	1.129 (0.262)	0.954 (0.102)	0.841
1418199974	16	vitamin K-dependent protein S [Canis lupus dingo]	0.96 (0.143)	1.026 (0.061)	0.310
1418200084	7	transferrin receptor protein 1 [Canis lupus dingo]	1.13 (0.046)	0.764 (0.206)	0.016
1418204343	2	C-C motif chemokine 14-like [Canis lupus dingo]	1.024 (0.018)	1.124 (0.096)	0.100
1418204633	13	phosphatidylinositol-glycan-specific phospholipase D isoform X2 [Canis lupus dingo]	0.913 (0.096)	1.137 (0.198)	0.063
1418205161	2	coagulation factor XIII A chain [Canis lupus dingo]	1.094 (0)	0.592 (0.582)	1.000
1418207941	10	plasma protease C1 inhibitor [Canis lupus dingo]	1.002 (0.163)	0.799 (0.211)	0.310
1418210682	3	actin, gamma-enteric smooth muscle [Canis lupus dingo]	0.878 (0.057)	0.865 (0.192)	0.690
1418212773	74	apolipoprotein B-100 [Canis lupus dingo]	0.932 (0.084)	0.782 (0.148)	0.063
1418213974	47	alpha-2-macroglobulin [Canis lupus dingo]	1.031 (0.076)	0.896 (0.157)	0.008
1418213976	58	pregnancy zone protein-like isoform X1 [Canis lupus dingo]	1.05 (0.097)	0.847 (0.235)	0.222
1418213978	59	pregnancy zone protein-like isoform X2 [Canis lupus dingo]	1.052 (0.098)	0.839 (0.231)	0.222
1418213980	51	pregnancy zone protein-like isoform X3 [Canis lupus dingo]	1.015 (0.05)	0.976 (0.218)	0.421
1418216228	4	inter-alpha-trypsin inhibitor heavy chain H3 isoform X1 [Canis lupus dingo]	0.993 (0.116)	0.814 (0.142)	0.151
1418216230	4	inter-alpha-trypsin inhibitor heavy chain H3 isoform X2 [Canis lupus dingo]	0.993 (0.116)	0.814 (0.142)	0.151
1418216242	21	inter-alpha-trypsin inhibitor heavy chain H1 [Canis lupus dingo]	1.124 (0.043)	0.881 (0.064)	0.032
1418218016	7	leucine-rich alpha-2-glycoprotein [Canis lupus dingo]	0.945 (0.042)	0.892 (0.204)	0.365
1418218430	8	complement C3 [Canis lupus dingo]	0.929 (0.086)	1.005 (0.027)	0.190
1418218853	5	complement C3-like [Canis lupus dingo]	0.874 (0.422)	0.888 (0.157)	0.556
1418220357	5	tetranectin [Canis lupus dingo]	1.153 (0.103)	0.941 (0.25)	0.310
1418221463	6	keratin, type II cytoskeletal 1 [Canis lupus dingo]	1.07 (0.285)	1.309 (4.301)	0.700
1418222274	13	hemoglobin subunit beta [Canis lupus dingo]	0.624 (0.137)	0.832 (0.169)	0.286
1418222276	8	hemoglobin subunit beta-like [Canis lupus dingo]	0.586 (0.165)	0.79 (0.242)	0.486
1418223194	7	serum amyloid A protein-like [Canis lupus dingo]	0.15 (0.57)	0.117 (0.176)	0.786
1418223200	4	serum amyloid A protein-like [Canis lupus dingo]	0.109 (0.411)	0.106 (0.117)	0.786
1418223204	7	serum amyloid A protein [Canis lupus dingo]	0.15 (0.57)	0.117 (0.176)	0.786

1418241889	5	apolipoprotein A-I [Canis lupus dingo]	1.206 (0.07)	1.18 (0.447)	0.310
1418241891	25	apolipoprotein A-IV [Canis lupus dingo]	0.937 (0.334)	1.097 (0.219)	0.690
1418243013	10	complement factor I isoform X1 [Canis lupus dingo]	1.065 (0.12)	0.986 (0.046)	0.286
1418243015	10	complement factor I isoform X2 [Canis lupus dingo]	1.065 (0.12)	0.986 (0.046)	0.286
1418243017	10	complement factor I isoform X3 [Canis lupus dingo]	1.065 (0.12)	0.986 (0.046)	0.286
1418243019	9	complement factor I isoform X4 [Canis lupus dingo]	1.05 (0.118)	0.986 (0.042)	0.286
1418245069	3	carboxypeptidase N catalytic chain isoform X2 [Canis lupus dingo]	0.961 (0.123)	0.954 (0.087)	0.905
1418245880	3	hyaluronan-binding protein 2 [Canis lupus dingo]	1.128 (0.185)	0.926 (0.362)	0.841
1418248118	4	actin, cytoplasmic 2 [Canis lupus dingo]	0.878 (0.057)	0.865 (0.192)	0.690
1418251736	5	LOW QUALITY PROTEIN: lipopolysaccharide-binding protein [Canis lupus dingo]	0.596 (0.162)	0.755 (2.247)	0.229
1418255638	7	plasma kallikrein isoform X1 [Canis lupus dingo]	1.074 (0.054)	1.083 (0.09)	0.730
1418256182	10	complement C1r subcomponent [Canis lupus dingo]	1.032 (0.041)	0.918 (0.182)	0.444
1418259722	4	thrombospondin-1 [Canis lupus dingo]	0.907 (0.101)	1.286 (0.284)	0.667
1418260227	2	uncharacterized protein LOC112676265 [Canis lupus dingo]	1.526 (0.246)	1 (0.128)	0.190
1418260262	2	uncharacterized protein LOC112676346 [Canis lupus dingo]	1.449 (0.618)	0.748 (0.406)	0.056
1418263577	3	immunoglobulin lambda-1 light chain-like [Canis lupus dingo]	1.306 (0.106)	0.768 (0.172)	0.008
1418264107	10	LOW QUALITY PROTEIN: heparin cofactor 2 [Canis lupus dingo]	0.86 (0.066)	0.947 (0.05)	0.310
1418266608	2	complement component C8 gamma chain isoform X1 [Canis lupus dingo]	0.959 (0.026)	1.081 (0.235)	0.730
1418267343	5	alpha-2-antiplasmin isoform X2 [Canis lupus dingo]	1 (0.181)	1.023 (0.12)	0.905
1418267345	4	alpha-2-antiplasmin isoform X3 [Canis lupus dingo]	1.044 (0.226)	1.036 (0.128)	0.730
1418267347	4	alpha-2-antiplasmin isoform X4 [Canis lupus dingo]	1.044 (0.226)	1.036 (0.128)	0.730
1418267349	5	alpha-2-antiplasmin isoform X1 [Canis lupus dingo]	1 (0.181)	1.023 (0.12)	0.905
1418267888	5	vitronectin [Canis lupus dingo]	0.966 (0.02)	1.008 (0.099)	0.841
1418275904	2	carboxypeptidase B2 [Canis lupus dingo]	0.758 (0.115)	0.882 (0.742)	1.000
1418290456	62	complement C4-A-like [Canis lupus dingo]	1.018 (0.072)	0.934 (0.044)	0.190
1418290462	7	complement C2 isoform X2 [Canis lupus dingo]	0.91 (0.011)	1.19 (0.385)	0.151
1418290466	27	complement factor B [Canis lupus dingo]	1.093 (0.052)	0.939 (0.129)	0.222
1418297237	13	alpha-1B-glycoprotein [Canis lupus dingo]	1.09 (0.016)	0.639 (0.367)	0.151
1418304648	3	apolipoprotein C-II [Canis lupus dingo]	1.044 (0.18)	1.5 (0.401)	0.310
1418304654	4	apolipoprotein C-I [Canis lupus dingo]	0.983 (0.234)	1.153 (0.214)	0.690
1418305616	2	xaa-Pro dipeptidase isoform X1 [Canis lupus dingo]	1.135 (0.047)	0.967 (0.072)	0.057

1418311120	3	complement component C8 alpha chain isoform X1 [Canis lupus dingo]	1.075 (0.112)	0.95 (0.32)	0.229
1418311122	3	complement component C8 alpha chain isoform X2 [Canis lupus dingo]	1.075 (0.112)	0.95 (0.32)	0.229
1418312041	2	microfibril-associated glycoprotein 4 [Canis lupus dingo]	0.99 (0.016)	0.945 (0.22)	0.700
1418312750	5	sex hormone-binding globulin isoform X1 [Canis lupus dingo]	1.258 (0.456)	1.403 (0.876)	0.629
1418314244	5	transthyretin [Canis lupus dingo]	0.914 (0.351)	1.071 (0.652)	0.841
1418315459	3	C4b-binding protein beta chain [Canis lupus dingo]	1.03 (0.126)	1.343 (0.146)	0.032
1418315462	24	C4b-binding protein alpha chain [Canis lupus dingo]	0.924 (0.137)	1.162 (0.096)	0.151
1418315968	3	sulfhydryl oxidase 1 isoform X1 [Canis lupus dingo]	0.906 (0.084)	0.763 (1.475)	1.000
1418315970	3	sulfhydryl oxidase 1 isoform X2 [Canis lupus dingo]	0.906 (0.084)	0.763 (1.475)	1.000
1418315972	3	sulfhydryl oxidase 1 isoform X3 [Canis lupus dingo]	0.906 (0.084)	0.763 (1.475)	1.000
1418316947	2	coagulation factor V [Canis lupus dingo]	0.894 (0.119)	1.087 (0.289)	0.556
1418317684	15	CD5 antigen-like [Canis lupus dingo]	1.07 (0.078)	1.071 (0.214)	1.000
1418321303	31	complement C5 [Canis lupus dingo]	0.922 (0.208)	0.938 (0.071)	0.905
1418322170	2	protein AMBP [Canis lupus dingo]	1.191 (0.091)	1.087 (0.169)	0.310
1418322411	15	gelsolin isoform X1 [Canis lupus dingo]	1.195 (0.109)	0.795 (0.103)	0.032
1418322413	15	gelsolin isoform X2 [Canis lupus dingo]	1.195 (0.109)	0.795 (0.103)	0.032
1418322415	15	gelsolin isoform X3 [Canis lupus dingo]	1.195 (0.109)	0.795 (0.103)	0.032
1418322788	8	alpha-1-acid glycoprotein 1-like [Canis lupus dingo]	0.562 (0.25)	0.556 (0.3)	0.686
1418324502	3	putative maltase-glucoamylase-like protein FLJ16351 [Canis lupus dingo]	0.901 (0.285)	1.018 (0.516)	1.000
1418324508	32	maltase-glucoamylase, intestinal isoform X1 [Canis lupus dingo]	0.83 (0.026)	1.061 (0.242)	0.016
1418324514	32	maltase-glucoamylase, intestinal isoform X2 [Canis lupus dingo]	0.83 (0.026)	1.061 (0.242)	0.016
1418325925	3	coagulation factor XII isoform X1 [Canis lupus dingo]	1.109 (0.141)	0.996 (0.159)	0.905
1418325927	3	coagulation factor XII isoform X2 [Canis lupus dingo]	1.109 (0.141)	0.996 (0.159)	0.905
1418325929	3	coagulation factor XII isoform X3 [Canis lupus dingo]	1.109 (0.141)	0.996 (0.159)	0.905
1418327084	2	mannose-binding protein C [Canis lupus dingo]	1.224 (0.284)	0.982 (0.496)	0.629
1418327853	7	glutathione peroxidase 3 [Canis lupus dingo]	1.161 (0.084)	1.127 (0.053)	0.730
1418328547	4	LOW QUALITY PROTEIN: serum albumin-like [Canis lupus dingo]	1.058 (0.086)	1.035 (0.051)	0.381
1418328657	26	vitamin D-binding protein [Canis lupus dingo]	1.033 (0.108)	1.067 (0.448)	0.651
1418328671	26	afamin [Canis lupus dingo]	1.07 (0.125)	0.909 (0.272)	0.095
1418329739	17	complement component C7 isoform X1 [Canis lupus dingo]	0.95 (0.154)	0.836 (0.034)	0.095
1418329741	16	complement component C7 isoform X2 [Canis lupus dingo]	0.947 (0.166)	0.826 (0.008)	0.151

1418329743	15	complement component C7 isoform X3 [Canis lupus dingo]	0.93 (0.135)	0.831 (0.063)	0.056
1418330062	6	complement component C9 [Canis lupus dingo]	0.859 (0.216)	1.012 (0.126)	0.548
1418330227	7	complement component C6 [Canis lupus dingo]	0.928 (0.178)	1.011 (0.32)	0.151
1418333042	2	actin, clone 302-like [Canis lupus dingo]	0.963 (0.086)	0.701 (0.408)	0.100
1418333238	2	hepatocyte growth factor activator [Canis lupus dingo]	0.823 (0.031)	0.874 (0)	0.667
1418336203	2	lumican [Canis lupus dingo]	1.081 (0.176)	0.97 (0.002)	1.000
1418336346	24	fibrinogen alpha chain [Canis lupus dingo]	0.431 (0.057)	0.44 (0.096)	0.421
1418336465	24	fibrinogen beta chain [Canis lupus dingo]	0.564 (0.104)	0.622 (0.113)	0.286
1418337563	6	keratin, type I cytoskeletal 10 isoform X1 [Canis lupus dingo]	1.127 (0.091)	1.057 (0.389)	0.730
1418337565	6	keratin, type I cytoskeletal 10 isoform X2 [Canis lupus dingo]	1.127 (0.091)	1.057 (0.389)	0.730
1418337567	6	keratin, type I cytoskeletal 10 isoform X3 [Canis lupus dingo]	1.127 (0.091)	1.057 (0.389)	0.730
1418340066	2	membrane primary amine oxidase isoform X2 [Canis lupus dingo]	0.89 (0.088)	1.036 (0.146)	0.063
1418340495	4	coagulation factor X-like isoform X1 [Canis lupus dingo]	1.038 (0.042)	1.014 (0.122)	0.629
1418341514	3	actin, aortic smooth muscle [Canis lupus dingo]	0.878 (0.057)	0.865 (0.192)	0.690
1418343247	57	fibronectin isoform X1 [Canis lupus dingo]	1.053 (0.092)	1.063 (0.199)	0.595
1418343249	57	fibronectin isoform X2 [Canis lupus dingo]	1.053 (0.092)	1.063 (0.199)	0.595
1418343251	57	fibronectin isoform X3 [Canis lupus dingo]	1.053 (0.092)	1.063 (0.199)	0.595
1418343253	58	fibronectin isoform X4 [Canis lupus dingo]	1.051 (0.094)	1.061 (0.225)	0.730
1418343255	58	fibronectin isoform X5 [Canis lupus dingo]	1.051 (0.094)	1.061 (0.225)	0.730
1418343257	56	fibronectin isoform X6 [Canis lupus dingo]	1.053 (0.093)	1.063 (0.199)	0.595
1418343259	58	fibronectin isoform X7 [Canis lupus dingo]	1.051 (0.094)	1.061 (0.225)	0.730
1418343261	54	fibronectin isoform X8 [Canis lupus dingo]	1.046 (0.098)	1.046 (0.18)	0.730
1418343263	58	fibronectin isoform X9 [Canis lupus dingo]	1.051 (0.094)	1.061 (0.225)	0.730
1418343265	56	fibronectin isoform X10 [Canis lupus dingo]	1.053 (0.093)	1.063 (0.199)	0.595
1418343267	57	fibronectin isoform X11 [Canis lupus dingo]	1.051 (0.095)	1.061 (0.225)	0.730
1418345970	11	alpha-1-antichymotrypsin [Canis lupus dingo]	0.775 (0.02)	0.867 (0.212)	0.343
1418346025	3	corticosteroid-binding globulin-like [Canis lupus dingo]	0.92 (0.107)	1.038 (0.325)	0.690
1418346186	6	plasma serine protease inhibitor [Canis lupus dingo]	0.875 (0.448)	0.86 (0.212)	0.889
1418502140	18	inter-alpha-trypsin inhibitor heavy chain H2 [Canis lupus dingo]	1.076 (0.112)	0.916 (0.15)	0.008
1418506879	2	complement C1q subcomponent subunit A [Canis lupus dingo]	0.931 (0.133)	1.57 (0.767)	0.286
1418507477	5	complement C1q subcomponent subunit B [Canis lupus dingo]	0.957 (0.035)	1.013 (0.062)	0.254

1418509163	3	actin, alpha skeletal muscle [Canis lupus dingo]	0.878 (0.057)	0.865 (0.192)	0.690
1418510222	26	ceruloplasmin isoform X1 [Canis lupus dingo]	0.869 (0.129)	0.845 (0.056)	1.000
1418510226	26	ceruloplasmin isoform X2 [Canis lupus dingo]	0.869 (0.129)	0.845 (0.056)	1.000
1418510228	26	ceruloplasmin isoform X3 [Canis lupus dingo]	0.869 (0.129)	0.845 (0.056)	1.000
1418510230	26	ceruloplasmin isoform X4 [Canis lupus dingo]	0.869 (0.129)	0.845 (0.056)	1.000
1418510423	2	serotransferrin-like [Canis lupus dingo]	0.903 (0.116)	0.851 (0.536)	1.000
1418515478	3	adiponectin isoform X1 [Canis lupus dingo]	1.212 (0.103)	0.585 (0.081)	0.151
1418515495	10	alpha-2-HS-glycoprotein [Canis lupus dingo]	0.972 (0.469)	1.034 (0.357)	0.310
1418515534	3	fetuin-B [Canis lupus dingo]	0.866 (0.318)	0.828 (0.437)	0.841
1418515616	13	histidine-rich glycoprotein isoform X1 [Canis lupus dingo]	1.019 (0.572)	1.306 (0.442)	1.000
1418515618	9	histidine-rich glycoprotein isoform X2 [Canis lupus dingo]	0.907 (0.344)	1.3 (0.585)	0.690
1483452888	2	TPA: adiponectin C [Canis lupus familiaris]	0.931 (0.133)	1.57 (0.767)	0.286

Table S4. Saliva proteins identified in dogs with diabetes mellitus (DM) and healthy controls (HC).

Accession Number	No. Unique Peptides	Protein Function	Median (IQR) Healthy	Median (IQR) DM	*p*
123511	16	RecName: Full=Haptoglobin; Contains: RecName: Full=Haptoglobin alpha chain; Contains: RecName: Full=Haptoglobin beta chain	0.56 (0.114)	0.832 (0.071)	0.111
124728	5	RecName: Full=Involucrin	1.002 (0.347)	0.853 (0.31)	1.000
124847	4	RecName: Full=Double-headed protease inhibitor, submandibular gland	0.259 (0.145)	0.453 (0.297)	0.111
163954	8	glycoprotein 80 [Canis lupus familiaris]	0.76 (0.398)	0.722 (0.547)	0.595
258499	14	haptoglobin heavy chain, HpH chain [dogs, Peptide, 245 aa]	0.585 (0.124)	0.836 (0.092)	0.111
264597	2	tissue kallikrein A beta-chain, CPK-A beta-chain {N-terminal} {EC 3.4.21.35} [dogs, pancreas, Peptide Partial, 38 aa]	2.251 (0.389)	1.072 (0.078)	0.151
264599	2	tissue kallikrein B beta-chain, CPK-B beta-chain {N-terminal} {EC 3.4.21.35} [dogs, pancreas, Peptide Partial, 39 aa]	2.251 (0.389)	1.072 (0.078)	0.151
494395	3	Chain C, Myeloperoxidase	0.949 (0.595)	1.023 (0.403)	0.421
598107	17	IgA heavy chain constant region, partial [Canis lupus familiaris]	1.266 (0.799)	0.894 (0.341)	0.310
624685	3	heat-shock protein [Canis lupus familiaris]	1.207 (0.52)	1.195 (0.257)	1.000
945179	3	CE1 [Canis lupus familiaris]	0.76 (0.089)	0.854 (0.021)	0.111
1222669	2	beta-actin, partial [Canis lupus familiaris]	0.919 (0.399)	0.742 (0.162)	0.413
1304047	2	fibrinogen A-alpha-chain, partial [Canis lupus familiaris]	0.316 (0.546)	1.231 (0.552)	0.095
1585652	2	platelet-activating factor acetylhydrolase	0.986 (0.574)	0.686 (0.303)	0.114

2564101	7	gelatinase B [Canis lupus familiaris]	0.694 (0.372)	0.896 (0.432)	0.690
3121745	13	RecName: Full=Major allergen Can f 1; AltName: Full=Allergen Dog 1; AltName: Allergen=Can f 1; Flags: Precursor	0.492 (0.041)	0.88 (0.461)	0.016
3121746	7	RecName: Full=Minor allergen Can f 2; AltName: Full=Allergen Dog 2; AltName: Allergen=Can f 2; Flags: Precursor	1.167 (0.36)	0.83 (0.185)	0.063
3413469	6	desmocollin type 2, partial [Canis lupus familiaris]	0.933 (0.208)	0.96 (0.289)	0.841
3915607	13	RecName: Full=Apolipoprotein A-I; Short=Apo-AI; Short=ApoA-I; AltName: Full=Apolipoprotein A1; Contains: RecName: Full=Proapolipoprotein A-I; Short=ProapoA-I; Contains: RecName: Full=Truncated apolipoprotein A-I; Flags: Precursor	0.42 (0.246)	0.546 (0.313)	0.905
4101628	3	desmoglein-1 precursor [Canis lupus familiaris]	0.794 (0.245)	1.155 (0.28)	0.229
4115557	2	catalase [Canis lupus familiaris]	0.698 (0.088)	0.88 (0)	0.667
4868451	3	type IV collagenase MMP-9, partial [Canis lupus familiaris]	0.782 (0.56)	0.896 (0.397)	0.421
5441519	3	ubiquitin-ribosomal protein L40 fusion protein [Canis lupus familiaris]	0.568 (0.384)	0.791 (0.201)	0.310
5597005	2	beta-actin [Canis lupus familiaris]	0.924 (0.351)	0.827 (0.183)	0.690
5822852	3	ubiquitin, partial [Canis lupus familiaris]	0.568 (0.384)	0.791 (0.201)	0.310
6687188	2	albumin [Canis lupus familiaris]	1.303 (0.846)	0.819 (0.241)	0.413
6983847	2	glyceraldehyde-3-phosphate dehydrogenase [Canis lupus familiaris]	1.058 (0.467)	0.824 (0.004)	0.016
7595920	2	glycogen phosphorylase, partial [Canis lupus familiaris]	0.732 (0.188)	0.946 (0.368)	0.229
8699209	4	cyclophilin A, partial [Canis lupus familiaris]	1.247 (0.087)	0.947 (0.641)	0.286
8928188	2	RecName: Full=Lysozyme C, milk isozyme; AltName: Full=1,4-beta-N-acetylmuramidase C	1.542 (0.85)	1.461 (1.239)	0.690
8928189	9	RecName: Full=Lysozyme C, spleen isozyme; AltName: Full=1,4-beta-N-acetylmuramidase C	0.918 (1.464)	0.829 (0.18)	0.690
9257149	2	Chain A, X-Ray Crystal Structure Analysis Of Canine Milk Lysozyme (Apo-Type)	1.542 (0.85)	1.461 (1.239)	0.690
11034716	7	matrix metalloproteinase-9 [Canis lupus familiaris]	0.694 (0.372)	0.896 (0.432)	0.690
12082093	2	catalase [Canis lupus familiaris]	0.698 (0.088)	0.88 (0)	0.667
13787135	2	Chain B, Lysozyme C	1.542 (0.85)	1.461 (1.239)	0.690
15419605	3	masticatory epithelia keratin 2p [Canis lupus familiaris]	0.329 (0.08)	0.465 (1.687)	0.700
16607663	5	unnamed protein product [Canis lupus familiaris]	0.755 (0.469)	0.724 (0.125)	0.690
16607675	4	unnamed protein product [Canis lupus familiaris]	0.755 (0.469)	0.724 (0.125)	0.690
16607718	5	unnamed protein product [Canis lupus familiaris]	0.755 (0.469)	0.724 (0.125)	0.690
16607721	6	unnamed protein product [Canis lupus familiaris]	0.68 (0.797)	0.813 (0.074)	1.000
17298186	3	heat shock protein 70 [Canis lupus familiaris]	0.593 (0.541)	1.057 (0.127)	0.310
18150346	5	Cu/Zn superoxide dismutase [Canis lupus familiaris]	0.996 (0.537)	0.933 (0.152)	1.000
19715661	2	immunoglobulin J chain, partial [Canis lupus familiaris]	0.841 (0.047)	0.913 (0.272)	0.690

29292272	7	precursor Can f II, partial [Canis lupus familiaris]	1.167 (0.36)	0.83 (0.185)	0.063
29292274	5	precursor Can f II, partial [Canis lupus familiaris]	2.459 (0.632)	0.981 (0.367)	0.032
39654740	2	Chain A, Endoplasmin	1.395 (0.83)	1.069 (0)	1.000
46392561	2	immunoglobulin alpha heavy chain constant regin variant A, partial [Canis lupus familiaris]	0.89 (1.683)	1.053 (0.092)	1.000
50542205	2	histone H4, partial [Canis lupus familiaris]	0.407 (0.41)	0.688 (0.172)	0.556
50978926	4	rab GDP dissociation inhibitor beta [Canis lupus familiaris]	1.137 (0.898)	0.984 (0.158)	0.700
50978992	6	matrix metalloproteinase-9 precursor [Canis lupus familiaris]	0.668 (0.373)	0.776 (0.432)	0.548
50979166	5	endoplasmin precursor [Canis lupus familiaris]	0.722 (0.594)	0.926 (0.44)	0.629
50979214	6	cathelicidin antimicrobial peptide precursor [Canis lupus familiaris]	0.68 (0.289)	1.106 (0.636)	0.310
50979246	4	neutrophil elastase precursor [Canis lupus familiaris]	0.444 (0.363)	0.541 (0.346)	0.548
55741639	8	kallikrein-1 precursor [Canis lupus familiaris]	1.934 (0.664)	0.858 (0.077)	0.190
56749085	3	RecName: Full=Heat shock 70 kDa protein 1	0.593 (0.541)	1.057 (0.127)	0.310
57088159	4	glutathione S-transferase Mu 3 isoform X2 [Canis lupus familiaris]	0.965 (0.328)	1.086 (0.144)	0.905
57089773	2	myosin regulatory light chain 12B [Canis lupus familiaris]	0.488 (0.483)	0.887 (0.149)	0.310
57090217	3	heat shock-related 70 kDa protein 2 [Canis lupus familiaris]	0.54 (0.219)	1.104 (0.268)	0.200
57092971	3	malate dehydrogenase, cytoplasmic [Canis lupus familiaris]	0.657 (0.118)	0.91 (0.123)	0.016
57099669	6	cofilin-1 [Canis lupus familiaris]	0.887 (0.541)	0.948 (0.136)	0.841
57104142	15	BPI fold-containing family B member 1 isoform X2 [Canis lupus familiaris]	0.65 (1.341)	0.653 (0.206)	1.000
57106959	7	rho GDP-dissociation inhibitor 2 [Canis lupus familiaris]	0.529 (0.214)	0.729 (0.429)	0.127
57106965	2	histone H2A.J [Canis lupus familiaris]	0.575 (0.406)	0.821 (0.058)	0.690
57109938	3	kininogen-1 isoform X2 [Canis lupus familiaris]	0.613 (0.586)	0.911 (0.392)	0.400
57110393	2	histone H2A type 1-A [Canis lupus familiaris]	0.575 (0.406)	0.821 (0.058)	0.690
60734606	6	unnamed protein product, partial [Canis lupus familiaris]	1.494 (0.522)	0.861 (0.217)	0.222
60734607	8	unnamed protein product, partial [Canis lupus familiaris]	1.203 (0.875)	0.958 (0.464)	0.310
62631867	5	heat shock protein Apg-2 [Canis lupus familiaris]	0.742 (0.445)	1.061 (0.287)	0.095
70794744	4	serine protease inhibitor Kazal-type 5 precursor [Canis lupus familiaris]	0.611 (0.143)	0.86 (0.254)	0.095
70909945	4	zinc alpha-2-glycoprotein 1, partial [Canis lupus familiaris]	1.296 (0.398)	0.883 (0.391)	0.229
71042092	2	Chain A, Endoplasmin	1.395 (0.83)	1.069 (0)	1.000
73945839	2	serpin B10 [Canis lupus familiaris]	1.088 (0.024)	0.721 (0.164)	0.100
73947736	12	alpha-actinin-4 isoform X4 [Canis lupus familiaris]	0.612 (0.362)	1.075 (0.033)	0.095
73948247	4	ly6/PLAUR domain-containing protein 3 [Canis lupus familiaris]	0.669 (0.117)	0.891 (0.301)	0.095

73948372	3	F-box only protein 50 [Canis lupus familiaris]	0.864 (0.363)	0.646 (0.374)	0.841
73952852	8	prosaposin isoform X3 [Canis lupus familiaris]	0.864 (0.188)	0.921 (0.3)	0.421
73956158	2	phosphoglucomutase-1 isoform X2 [Canis lupus familiaris]	0.737 (0.376)	0.897 (0.216)	0.714
73957095	16	haptoglobin-like [Canis lupus familiaris]	0.56 (0.114)	0.832 (0.071)	0.111
73959451	4	zymogen granule protein 16 homolog B [Canis lupus familiaris]	3.775 (3.763)	0.929 (0.422)	0.222
73963050	3	proteasome subunit alpha type-3 [Canis lupus familiaris]	0.303 (0)	0.887 (0.149)	0.667
73963339	5	alpha-actinin-1 isoform X4 [Canis lupus familiaris]	0.809 (0.361)	1.001 (0.08)	0.095
73963357	5	alpha-actinin-1 isoform X5 [Canis lupus familiaris]	0.809 (0.361)	1.001 (0.08)	0.095
73964747	3	rho GDP-dissociation inhibitor 1 [Canis lupus familiaris]	0.685 (0.183)	1.067 (0.462)	0.310
73964749	7	protein disulfide-isomerase [Canis lupus familiaris]	0.904 (0.117)	0.985 (0.159)	0.310
73967156	5	14-3-3 protein epsilon isoform X2 [Canis lupus familiaris]	0.672 (0.254)	1.135 (0.17)	0.032
73977992	6	fibrinogen gamma chain isoform X2 [Canis lupus familiaris]	0.371 (0.274)	0.945 (0.046)	0.151
73978329	5	fibrinogen alpha chain [Canis lupus familiaris]	0.402 (0.399)	0.831 (0.375)	0.222
73978762	9	prolactin-inducible protein [Canis lupus familiaris]	1.399 (0.548)	0.927 (0.229)	0.310
73980076	7	xanthine dehydrogenase/oxidase isoform X1 [Canis lupus familiaris]	0.786 (0.422)	1.066 (0.232)	0.222
73981516	2	histone H2A type 2-A [Canis lupus familiaris]	0.575 (0.406)	0.821 (0.058)	0.690
73988725	7	hemopexin [Canis lupus familiaris]	0.603 (0.333)	0.852 (0.132)	0.151
73990367	3	ceruloplasmin isoform X3 [Canis lupus familiaris]	0.464 (0.397)	1.028 (0.074)	0.111
73991635	3	adenosylhomocysteinase [Canis lupus familiaris]	0.383 (0.084)	0.878 (0.26)	0.100
73992235	2	BPI fold-containing family A member 1 [Canis lupus familiaris]	1.137 (0.4)	0.835 (0.003)	1.000
73995130	3	polyubiquitin-C [Canis lupus familiaris]	0.568 (0.384)	0.791 (0.201)	0.310
73996461	2	keratin, type II cytoskeletal 78 [Canis lupus familiaris]	0.128 (0)	2.51 (1.576)	0.667
74003556	3	fetuin-B [Canis lupus familiaris]	0.543 (0.633)	0.979 (0.527)	0.548
74003808	3	ribosyldihydronicotinamide dehydrogenase [quinone] [Canis lupus familiaris]	0.962 (0.356)	0.879 (0.1)	1.000
74004170	2	histone H2A type 1-E-like [Canis lupus familiaris]	0.575 (0.406)	0.821 (0.058)	0.690
74007807	4	phosphoglycerate kinase 1 [Canis lupus familiaris]	0.522 (0.276)	0.976 (0.344)	0.222
74008809	6	filamin-A [Canis lupus familiaris]	0.464 (0.437)	1.163 (0.117)	0.032
75062693	4	RecName: Full=Keratin, type II cytoskeletal 1; AltName: Full=Cytokeratin-1; Short=CK-1; AltName: Full=Epithelial keratin-1; AltName: Full=Keratin-1; Short=K1; AltName: Full=Type-II keratin Kb1	0.419 (0.084)	0.562 (0.19)	0.063
76363530	12	RecName: Full=Triosephosphate isomerase; Short=TIM; AltName: Full=Methylglyoxal synthase; AltName: Full=Triose-phosphate isomerase	0.851 (0.194)	0.985 (0.113)	0.032
84872712	8	lactoferrin, partial [Canis lupus familiaris]	1.435 (0.278)	0.877 (0.507)	0.175

89574135	3	mitochondrial malate dehydrogenase 2, NAD, partial [Canis lupus familiaris]	0.575 (0.144)	1.05 (0.224)	0.008
103484123	4	globin, partial [Canis lupus familiaris]	1.119 (2.661)	0.783 (0.937)	0.548
114326321	4	phosphatidylethanolamine-binding protein 1 [Canis lupus familiaris]	0.818 (0.303)	0.896 (0.107)	0.413
116667120	2	Chain A, Endoplasmin	1.395 (0.83)	1.069 (0)	1.000
119637732	6	alpha 1 antitrypsin [Canis lupus familiaris]	1.07 (1.216)	0.723 (0.536)	0.548
122135145	21	RecName: Full=Myosin-9; AltName: Full=Myosin heavy chain 9; AltName: Full=Myosin heavy chain, non-muscle IIa; AltName: Full=Non-muscle myosin heavy chain IIa; Short=NMMHC II-a; Short=NMMHC-IIA	0.522 (0.449)	1.1 (0.424)	0.151
124390009	2	immunoglobulin heavy chain constant region CH2, partial [Canis lupus familiaris]	0.699 (0.464)	0.763 (0.062)	0.690
124390013	2	immunoglobulin heavy chain constant region CH4, partial [Canis lupus familiaris]	1.691 (0.733)	1.001 (0.204)	0.151
146743249	2	immunoglobulin mu heavy chain variable region, partial [Canis lupus familiaris]	1.691 (0.733)	1.001 (0.204)	0.151
156875888	2	trefoil factor family peptide 3 [Canis lupus familiaris]	1.047 (0.244)	0.876 (0.176)	0.556
157061758	2	trefoil factor family peptide 3, partial [Canis lupus]	1.047 (0.244)	0.876 (0.176)	0.556
158430851	2	Chain A, Glutamine Synthetase	0.455 (0.791)	0.921 (0.441)	0.548
158936956	4	protease inhibitor [Canis lupus familiaris]	1.253 (0.147)	0.908 (0.354)	0.222
159794950	3	Chain G, Endoplasmin	0.648 (0.478)	0.888 (0.44)	0.400
159794957	5	Chain B, Endoplasmin	0.722 (0.594)	0.926 (0.44)	0.629
159794959	5	Chain B, Endoplasmin	0.722 (0.594)	0.926 (0.44)	0.629
164499359	3	secretory leukocyte peptidase inhibitor, partial [Canis lupus familiaris]	1.204 (0.36)	0.922 (0.155)	0.222
195546936	3	WD repeat-containing protein 1 [Canis lupus familiaris]	0.718 (0.496)	1.068 (0)	1.000
208342038	2	immunoglobulin heavy chain variable region, partial [Canis lupus familiaris]	0.802 (0.623)	0.812 (0.422)	1.000
223556019	2	carbonic anhydrase 2 [Canis lupus familiaris]	0.518 (0.723)	0.9 (0.387)	0.548
224969390	7	S100 calcium binding protein A8 [Canis lupus familiaris]	0.333 (0.423)	1.357 (0.508)	0.056
227343817	15	Chain B, Crystal Structure Of Dog (Canis Familiaris) Hemoglobin	0.492 (0.258)	0.496 (0.086)	1.000
296863542	7	Chain A, Crystal Structure Of The Dog Lipocalin Allergen Can F 2 And Implications For Cross-Reactivity To The Cat Allergen Fel D 4	1.167 (0.36)	0.83 (0.185)	0.063
305657831	3	metavinculin variant, partial [Canis lupus familiaris]	0.283 (0.266)	1.269 (0.361)	0.200
339305349	2	lactotransferrin, partial [Canis lupus familiaris]	0.854 (0.56)	0.945 (0.185)	1.000
339305351	2	lactotransferrin, partial [Canis lupus familiaris]	1.187 (0.318)	0.902 (0.076)	0.190
339305353	2	lactotransferrin, partial [Canis lupus familiaris]	1.246 (0.671)	1.074 (0.724)	0.548
345776590	3	myosin light polypeptide 6 isoform X1 [Canis lupus familiaris]	0.466 (0.734)	0.96 (0.387)	0.310
345778725	2	lactoylglutathione lyase [Canis lupus familiaris]	0.657 (0.185)	0.811 (0.259)	0.690

345779658	6	submaxillary gland androgen-regulated protein 3A isoform X2 [Canis lupus familiaris]	0.626 (0.353)	1.037 (0.581)	0.310
345779666	6	immunoglobulin J chain [Canis lupus familiaris]	1.23 (0.492)	1.002 (0.047)	0.548
345784150	4	actin-related protein 3 [Canis lupus familiaris]	0.515 (0.46)	1.048 (0.597)	0.151
345784333	5	serpin B5 isoform X1 [Canis lupus familiaris]	0.651 (0.42)	1.167 (0.182)	0.200
345787749	6	calreticulin [Canis lupus familiaris]	0.709 (0.035)	0.63 (0.014)	0.057
345788999	7	ceruloplasmin-like [Canis lupus familiaris]	0.529 (0.568)	0.839 (0.23)	0.190
345790559	3	phosphatidylethanolamine-binding protein 4 isoform X6 [Canis lupus familiaris]	0.924 (0.112)	1.051 (0.284)	0.700
345790561	3	phosphatidylethanolamine-binding protein 4 isoform X1 [Canis lupus familiaris]	0.924 (0.112)	1.051 (0.284)	0.700
345791839	6	keratin, type II cytoskeletal 6A isoform X2 [Canis lupus familiaris]	0.513 (0.174)	0.838 (1.171)	0.629
345792424	3	alpha-2-macroglobulin [Canis lupus familiaris]	0.457 (0.77)	1.049 (0.212)	0.548
345792633	3	phosphoglycerate mutase 1 [Canis lupus familiaris]	1.177 (0.023)	0.975 (0.359)	0.190
345794456	2	SH3 domain-binding glutamic acid-rich-like protein 3 [Canis lupus familiaris]	1.154 (0.397)	0.943 (0.285)	1.000
345796419	3	kininogen-1 isoform X1 [Canis lupus familiaris]	0.613 (0.586)	0.911 (0.392)	0.400
345797882	3	transgelin-2 [Canis lupus familiaris]	0.988 (0.606)	0.669 (0.2)	0.700
345798353	2	protein S100-P [Canis lupus familiaris]	0.642 (0.425)	0.837 (0.44)	0.222
345798988	5	vinculin [Canis lupus familiaris]	0.338 (0.266)	1.211 (0.344)	0.200
345799783	4	neutrophil collagenase [Canis lupus familiaris]	0.759 (0.252)	1.073 (0.032)	0.190
345800677	14	LOW QUALITY PROTEIN: alpha-enolase [Canis lupus familiaris]	0.59 (0.246)	1.069 (0.073)	0.095
345801939	5	coronin-1A [Canis lupus familiaris]	0.598 (0.548)	1.075 (0.312)	0.151
345803346	2	myosin regulatory light polypeptide 9 isoform X2 [Canis lupus familiaris]	0.488 (0.483)	0.887 (0.149)	0.310
345805399	3	keratin, type I cytoskeletal 24 [Canis lupus familiaris]	0.28 (0)	2.241 (1.796)	0.667
345806081	5	78 kDa glucose-regulated protein [Canis lupus familiaris]	0.873 (0.493)	0.9 (0.366)	0.421
356461040	4	L-lactate dehydrogenase B chain [Canis lupus familiaris]	0.914 (0.102)	0.77 (0.094)	0.114
356582247	3	NPC intracellular cholesterol transporter 2 precursor [Canis lupus familiaris]	0.76 (0.089)	0.854 (0.021)	0.111
356582259	2	prothymosin alpha [Canis lupus familiaris]	0.432 (0.155)	1.056 (0.286)	0.032
356582340	3	ubiquitin-40S ribosomal protein S27a [Canis lupus familiaris]	0.568 (0.384)	0.791 (0.201)	0.310
359318588	2	myosin-14 isoform X1 [Canis lupus familiaris]	0.792 (0.556)	1.332 (0)	1.000
359319594	3	coactosin-like protein, partial [Canis lupus familiaris]	0.873 (0.817)	1.168 (0.391)	0.690
359320010	2	antithrombin-III [Canis lupus familiaris]	0.32 (0.275)	0.766 (0.274)	0.343
359321687	2	histone H2A type 2-C [Canis lupus familiaris]	0.575 (0.406)	0.821 (0.058)	0.690
359321930	3	mammaglobin-A isoform X2 [Canis lupus familiaris]	0.384 (1.111)	0.416 (0.051)	0.905

359321944	7	transaldolase [Canis lupus familiaris]	0.781 (0.355)	0.985 (0.143)	0.310
359322579	2	acetyl-coenzyme A synthetase 2-like, mitochondrial [Canis lupus familiaris]	0.613 (0.155)	1.522 (0.125)	0.057
545487024	3	alpha-1B-glycoprotein [Canis lupus familiaris]	0.932 (0.541)	0.498 (0.038)	0.016
545492085	3	F-actin-capping protein subunit beta isoform X4 [Canis lupus familiaris]	0.501 (0)	0.925 (0.168)	0.667
545492660	2	calpastatin isoform X1 [Canis lupus familiaris]	0.684 (0.651)	0.898 (0.742)	0.629
545492662	2	calpastatin isoform X4 [Canis lupus familiaris]	0.684 (0.651)	0.898 (0.742)	0.629
545495176	8	prosaposin isoform X1 [Canis lupus familiaris]	0.864 (0.188)	0.921 (0.3)	0.421
545495178	8	prosaposin isoform X2 [Canis lupus familiaris]	0.864 (0.188)	0.921 (0.3)	0.421
545495181	8	prosaposin isoform X4 [Canis lupus familiaris]	0.864 (0.188)	0.921 (0.3)	0.421
545495183	8	prosaposin isoform X5 [Canis lupus familiaris]	0.864 (0.188)	0.921 (0.3)	0.421
545497049	8	heat shock cognate 71 kDa protein [Canis lupus familiaris]	0.585 (0.39)	1.081 (0.175)	0.032
545497152	2	histone H2AX [Canis lupus familiaris]	0.575 (0.406)	0.821 (0.058)	0.690
545500084	10	NAD(P)H dehydrogenase [quinone] 1 [Canis lupus familiaris]	0.632 (0.338)	0.838 (0.394)	0.548
545500782	4	actin, cytoplasmic 1 isoform X1 [Canis lupus familiaris]	1.145 (0.154)	0.76 (0.14)	0.190
545502107	5	myosin-11 isoform X2 [Canis lupus familiaris]	0.774 (0.459)	1.235 (0.724)	0.222
545504019	3	desmoglein-1 isoform X1 [Canis lupus familiaris]	0.794 (0.245)	1.155 (0.28)	0.229
545504091	2	tropomyosin alpha-3 chain isoform X6 [Canis lupus familiaris]	0.348 (0.844)	0.971 (0.028)	0.548
545506022	6	desmocollin-2 isoform X2 [Canis lupus familiaris]	0.933 (0.208)	0.96 (0.289)	0.841
545506026	6	desmocollin-2 isoform X4 [Canis lupus familiaris]	0.933 (0.208)	0.96 (0.289)	0.841
545507351	6	L-lactate dehydrogenase A chain-like [Canis lupus familiaris]	1.16 (0.692)	1.076 (0.535)	0.548
545510194	2	nucleoside diphosphate kinase A isoform X1 [Canis lupus familiaris]	0.586 (0.451)	1.019 (0.274)	0.190
545510196	3	nucleoside diphosphate kinase B isoform X1 [Canis lupus familiaris]	0.547 (0.248)	1.052 (0.139)	0.016
545511022	3	puromycin-sensitive aminopeptidase isoform X1 [Canis lupus familiaris]	0.523 (0.246)	1.047 (0.058)	0.100
545511447	10	myeloperoxidase [Canis lupus familiaris]	0.435 (0.543)	1.013 (0.449)	0.151
545514351	5	angiopoietin-related protein 5-like [Canis lupus familiaris]	1.708 (1.262)	1.082 (0.512)	0.056
545516431	5	heat shock 70 kDa protein 4 isoform X2 [Canis lupus familiaris]	0.742 (0.445)	1.061 (0.287)	0.095
545517912	6	prostaglandin reductase 1 [Canis lupus familiaris]	0.521 (0.506)	0.988 (0.26)	0.421
545518207	2	platelet-activating factor acetylhydrolase isoform X1 [Canis lupus familiaris]	0.986 (0.574)	0.686 (0.303)	0.114
545518209	2	platelet-activating factor acetylhydrolase isoform X2 [Canis lupus familiaris]	0.986 (0.574)	0.686 (0.303)	0.114
545519428	6	cysteine-rich secretory protein 2 isoform X1 [Canis lupus familiaris]	1.093 (0.57)	1.085 (0.254)	0.841
545519773	2	NADP-dependent malic enzyme [Canis lupus familiaris]	0.668 (0.31)	0.788 (0)	1.000

545521932	2	histone H2A type 3 [Canis lupus familiaris]	0.575 (0.406)	0.821 (0.058)	0.690
545524897	6	fibrinogen gamma chain isoform X1 [Canis lupus familiaris]	0.371 (0.274)	0.945 (0.046)	0.151
545527502	7	xanthine dehydrogenase/oxidase isoform X2 [Canis lupus familiaris]	0.786 (0.422)	1.066 (0.232)	0.222
545528000	6	macrophage-capping protein isoform X1 [Canis lupus familiaris]	0.63 (0.355)	0.993 (0.16)	0.222
545528998	5	involucrin [Canis lupus familiaris]	1.002 (0.347)	0.853 (0.31)	1.000
545531237	2	secretoglobin family 1D member 2-like [Canis lupus familiaris]	0.503 (0.134)	0.317 (0.398)	0.905
545533393	25	transketolase [Canis lupus familiaris]	0.648 (0.397)	1.061 (0.086)	0.032
545533633	9	hyaluronidase-1 isoform X1 [Canis lupus familiaris]	0.886 (0.983)	0.692 (0.136)	0.222
545536994	10	L-lactate dehydrogenase A chain isoform X2 [Canis lupus familiaris]	0.634 (0.51)	0.899 (0.26)	0.310
545537421	17	plastin-2 isoform X2 [Canis lupus familiaris]	0.538 (0.622)	1.111 (0.161)	0.016
545540364	15	BPI fold-containing family B member 1 isoform X1 [Canis lupus familiaris]	0.65 (1.341)	0.653 (0.206)	1.000
545545386	6	keratin, type II cytoskeletal 6A isoform X1 [Canis lupus familiaris]	0.513 (0.174)	0.838 (1.171)	0.629
545546412	6	pregnancy zone protein-like isoform X1 [Canis lupus familiaris]	0.826 (0.409)	1.155 (0.044)	0.229
545546414	6	pregnancy zone protein-like isoform X2 [Canis lupus familiaris]	0.826 (0.409)	1.155 (0.044)	0.229
545550333	6	pyruvate kinase PKM isoform X3 [Canis lupus familiaris]	1.026 (0.328)	0.822 (0.018)	0.730
545554572	2	histone H2A type 1-C [Canis lupus familiaris]	0.575 (0.406)	0.821 (0.058)	0.690
545558304	6	moesin isoform X1 [Canis lupus familiaris]	1.138 (0.648)	0.965 (0.011)	1.000
550600200	3	RecName: Full=Cadherin-1; AltName: Full=Epithelial cadherin; Short=E-cadherin; AltName: Full=Uvomorulin; AltName: CD_antigen=CD324; Contains: RecName: Full=E-Cad/CTF1; Contains: RecName: Full=E-Cad/CTF2; Contains: RecName: Full=E-Cad/CTF3; Flags: Precursor	0.497 (0.044)	0.872 (0.35)	0.100
558695388	2	plasminogen precursor [Canis lupus familiaris]	0.448 (0.282)	0.793 (0)	0.667
558695394	9	annexin A1 [Canis lupus familiaris]	0.566 (0.244)	0.873 (0.234)	0.222
558757359	9	ezrin [Canis lupus familiaris]	0.767 (0.14)	0.907 (0.167)	0.222
559098393	2	vimentin [Canis lupus familiaris]	0.762 (0.43)	0.838 (0.156)	0.857
559767198	2	H2A histone family, member Z [Canis lupus familiaris]	0.575 (0.406)	0.821 (0.058)	0.690
559767226	35	lactotransferrin precursor [Canis lupus familiaris]	0.933 (0.405)	0.896 (0.08)	0.556
560879429	4	zinc-alpha-2-glycoprotein precursor [Canis lupus familiaris]	1.296 (0.398)	0.883 (0.391)	0.229
562155348	13	glycogen phosphorylase, liver form [Canis lupus familiaris]	0.706 (0.378)	1.074 (0.252)	0.151
635545472	2	glutathione S-transferase alpha 3 [Canis lupus familiaris]	0.4 (0.681)	0.963 (0.584)	0.629
648216006	2	glutamine synthetase isoform 2 [Canis lupus familiaris]	0.455 (0.791)	0.921 (0.441)	0.548
648216199	2	glutamine synthetase isoform 1 [Canis lupus familiaris]	0.455 (0.791)	0.921 (0.441)	0.548

649656024	2	glutathione s-transferase alpha 3 [Canis lupus familiaris]	0.4 (0.681)	0.963 (0.584)	0.629
665505916	9	lysozyme C precursor [Canis lupus familiaris]	0.918 (1.464)	0.829 (0.18)	0.690
672890024	5	heat shock protein 90 kDa beta member 1, partial [Canis lupus familiaris]	0.722 (0.594)	0.926 (0.44)	0.629
696633650	2	heat shock protein 27, partial [Canis lupus familiaris]	1.207 (0.52)	1.195 (0.257)	1.000
922059102	3	heat shock protein 90 kDa alpha class B member 1, partial [Canis lupus familiaris]	0.619 (0.69)	0.937 (0.182)	0.548
922664320	2	uteroglobin, partial [Canis lupus familiaris]	1.817 (0)	0.402 (0.088)	0.667
924183513	4	actin, cytoplasmic 1 [Canis lupus familiaris]	1.145 (0.154)	0.76 (0.14)	0.190
924442847	4	actin, cytoplasmic 2 [Canis lupus familiaris]	1.145 (0.154)	0.76 (0.14)	0.190
924442944	3	heat shock protein beta-1 [Canis lupus familiaris]	1.207 (0.52)	1.195 (0.257)	1.000
925114454	2	lysozyme C, milk isozyme-like precursor [Canis lupus familiaris]	1.542 (0.85)	1.461 (1.239)	0.690
925115133	2	glyceraldehyde-3-phosphate dehydrogenase [Canis lupus familiaris]	1.058 (0.467)	0.824 (0.004)	0.016
928125111	13	LOW QUALITY PROTEIN: alpha-enolase-like [Canis lupus familiaris]	0.586 (0.252)	1.032 (0.034)	0.190
928128985	2	F-actin-capping protein subunit beta isoform X1 [Canis lupus familiaris]	0.501 (0)	0.925 (0.168)	0.667
928128987	3	F-actin-capping protein subunit beta isoform X2 [Canis lupus familiaris]	0.501 (0)	0.925 (0.168)	0.667
928129121	2	EF-hand domain-containing protein D2 [Canis lupus familiaris]	0.543 (0.357)	0.913 (0.412)	0.229
928129389	2	calpastatin isoform X2 [Canis lupus familiaris]	0.684 (0.651)	0.898 (0.742)	0.629
928129391	2	calpastatin isoform X3 [Canis lupus familiaris]	0.684 (0.651)	0.898 (0.742)	0.629
928129394	2	calpastatin isoform X5 [Canis lupus familiaris]	0.684 (0.651)	0.898 (0.742)	0.629
928129396	2	calpastatin isoform X6 [Canis lupus familiaris]	0.684 (0.651)	0.898 (0.742)	0.629
928129398	2	calpastatin isoform X9 [Canis lupus familiaris]	0.684 (0.651)	0.898 (0.742)	0.629
928129400	2	calpastatin isoform X10 [Canis lupus familiaris]	0.684 (0.651)	0.898 (0.742)	0.629
928129402	2	calpastatin isoform X11 [Canis lupus familiaris]	0.684 (0.651)	0.898 (0.742)	0.629
928129404	2	calpastatin isoform X12 [Canis lupus familiaris]	0.684 (0.651)	0.898 (0.742)	0.629
928129406	2	calpastatin isoform X14 [Canis lupus familiaris]	0.684 (0.651)	0.898 (0.742)	0.629
928129408	2	calpastatin isoform X15 [Canis lupus familiaris]	0.684 (0.651)	0.898 (0.742)	0.629
928129410	2	calpastatin isoform X16 [Canis lupus familiaris]	0.684 (0.651)	0.898 (0.742)	0.629
928129412	2	calpastatin isoform X18 [Canis lupus familiaris]	0.684 (0.651)	0.898 (0.742)	0.629
928129414	2	calpastatin isoform X19 [Canis lupus familiaris]	0.684 (0.651)	0.898 (0.742)	0.629
928129416	2	calpastatin isoform X17 [Canis lupus familiaris]	0.684 (0.651)	0.898 (0.742)	0.629
928131428	2	succinyl-CoA:3-ketoacid coenzyme A transferase 1, mitochondrial [Canis lupus familiaris]	0.468 (0.196)	0.981 (0.005)	0.333
928134045	4	beta-enolase isoform X1 [Canis lupus familiaris]	0.716 (0.569)	1.285 (0.668)	0.310

928134337	4	myosin-10 isoform X2 [Canis lupus familiaris]	1.162 (0.713)	1.036 (0.399)	0.794
928134341	4	myosin-10 isoform X4 [Canis lupus familiaris]	1.162 (0.713)	1.036 (0.399)	0.794
928134343	4	myosin-10 isoform X5 [Canis lupus familiaris]	1.162 (0.713)	1.036 (0.399)	0.794
928134704	2	phosphoglucomutase-1 isoform X1 [Canis lupus familiaris]	0.737 (0.376)	0.897 (0.216)	0.714
928137006	2	apoptosis-associated speck-like protein containing a CARD isoform X1 [Canis lupus familiaris]	1.274 (0.318)	1.73 (0.602)	0.667
928140605	9	protein S100-A9 [Canis lupus familiaris]	0.254 (0.442)	1.327 (0.417)	0.032
928141642	2	cathepsin G [Canis lupus familiaris]	0.885 (0.489)	1.047 (0)	1.000
928142969	8	heat shock protein HSP 90-alpha [Canis lupus familiaris]	0.479 (0.251)	0.83 (0.097)	0.151
928144438	6	keratin, type I cytoskeletal 10 isoform X1 [Canis lupus familiaris]	1.085 (0.887)	0.999 (0.182)	0.905
928144440	6	keratin, type I cytoskeletal 10 isoform X2 [Canis lupus familiaris]	1.085 (0.887)	0.999 (0.182)	0.905
928144442	6	keratin, type I cytoskeletal 10 isoform X3 [Canis lupus familiaris]	1.085 (0.887)	0.999 (0.182)	0.905
928151832	8	14-3-3 protein zeta/delta [Canis lupus familiaris]	0.896 (0.104)	1.068 (0.273)	0.690
928153482	2	LOW QUALITY PROTEIN: glyceraldehyde-3-phosphate dehydrogenase-like [Canis lupus familiaris]	1.058 (0.467)	0.824 (0.004)	0.016
928156540	2	maltase-glucoamylase, intestinal isoform X2 [Canis lupus familiaris]	0.406 (0)	1.22 (0.524)	0.667
928158112	4	macrophage-capping protein isoform X2 [Canis lupus familiaris]	0.514 (0.407)	1.03 (0.294)	0.095
928158750	2	protein S100-A11 [Canis lupus familiaris]	0.396 (0.246)	1.136 (0.528)	0.032
928162268	4	actin-related protein 2/3 complex subunit 4 [Canis lupus familiaris]	0.246 (0.384)	1.161 (0.118)	0.016
928165121	2	folate receptor beta isoform X2 [Canis lupus familiaris]	0.39 (0)	1.207 (0.605)	0.667
928167527	3	ceruloplasmin isoform X1 [Canis lupus familiaris]	0.464 (0.397)	1.028 (0.074)	0.111
928167632	2	serotransferrin [Canis lupus familiaris]	0.624 (0.489)	1.07 (0.098)	0.063
928174762	4	fatty acid-binding protein, epidermal [Canis lupus familiaris]	0.792 (0.078)	1.114 (0.593)	0.421
928175938	12	deleted in malignant brain tumors 1 protein isoform X1 [Canis lupus familiaris]	0.986 (0.363)	0.926 (0.273)	0.421
928175949	12	deleted in malignant brain tumors 1 protein isoform X2 [Canis lupus familiaris]	0.986 (0.363)	0.926 (0.273)	0.421
928175953	12	deleted in malignant brain tumors 1 protein isoform X3 [Canis lupus familiaris]	0.986 (0.363)	0.926 (0.273)	0.421
928180889	2	histone H2A-beta, sperm-like [Canis lupus familiaris]	0.575 (0.406)	0.821 (0.058)	0.690
928180938	2	histone H2A type 1-H [Canis lupus familiaris]	0.575 (0.406)	0.821 (0.058)	0.690
928180958	3	LOW QUALITY PROTEIN: uncharacterized protein LOC488306 [Canis lupus familiaris]	0.407 (0.41)	0.741 (0.166)	0.310
928181068	3	uncharacterized protein LOC100856160 [Canis lupus familiaris]	0.407 (0.41)	0.741 (0.166)	0.310
928182507	5	fibronectin isoform X4 [Canis lupus familiaris]	0.785 (0.703)	1.041 (0.501)	0.629
928182509	5	fibronectin isoform X5 [Canis lupus familiaris]	0.785 (0.703)	1.041 (0.501)	0.629
928182511	5	fibronectin isoform X7 [Canis lupus familiaris]	0.785 (0.703)	1.041 (0.501)	0.629

928182513	5	fibronectin isoform X9 [Canis lupus familiaris]	0.785 (0.703)	1.041 (0.501)	0.629
928182519	5	fibronectin isoform X11 [Canis lupus familiaris]	0.785 (0.703)	1.041 (0.501)	0.629
928182521	4	fibronectin isoform X1 [Canis lupus familiaris]	0.785 (0.703)	1.041 (0.501)	0.629
928182523	4	fibronectin isoform X10 [Canis lupus familiaris]	0.785 (0.703)	1.041 (0.501)	0.629
928183828	2	LOW QUALITY PROTEIN: ubiquitin-like modifier-activating enzyme 1 [Canis lupus familiaris]	0.781 (0.318)	1.041 (0)	1.000
928185024	6	moesin isoform X3 [Canis lupus familiaris]	1.138 (0.648)	0.965 (0.011)	1.000
928186182	2	histone H2A.V isoform X1 [Canis lupus familiaris]	0.575 (0.406)	0.821 (0.058)	0.690
928186186	2	histone H2A.V isoform X3 [Canis lupus familiaris]	0.575 (0.406)	0.821 (0.058)	0.690
928186528	2	mammaglobin-A-like [Canis lupus familiaris]	0.341 (0.188)	0.397 (0.138)	0.730
928186547	3	deleted in malignant brain tumors 1 protein-like, partial [Canis lupus familiaris]	1.128 (0.679)	0.908 (0.387)	0.222
1068388058	3	keratin, type II cytoskeletal 2 oral [Canis lupus familiaris]	0.329 (0.08)	0.465 (1.687)	0.700
1069415302	4	keratin, type II cytoskeletal 5 [Canis lupus familiaris]	0.253 (0.153)	0.43 (0.077)	0.190
1077167933	6	keratin, type I cytoskeletal 13 [Canis lupus familiaris]	0.251 (0.062)	0.401 (0.142)	0.190
1101972892	9	TPA: globin A1 [Canis lupus familiaris]	0.532 (0.336)	0.489 (0.1)	1.000
1104685307	2	Chain A, Serum Albumin	1.303 (0.846)	0.819 (0.241)	0.413
1239883019	3	peptidyl-prolyl cis-trans isomerase A [Canis lupus familiaris]	1 (0.115)	1.004 (0.683)	1.000
1239883228	3	uridine phosphorylase 1-like [Canis lupus familiaris]	0.408 (0.46)	1.007 (0.136)	0.400
1239883349	4	mammaglobin-B-like isoform X2 [Canis lupus familiaris]	0.384 (1.111)	0.416 (0.051)	0.905
1239884102	4	phospholipase A2 inhibitor and Ly6/PLAUR domain-containing protein, partial [Canis lupus familiaris]	2.268 (1.516)	1.329 (0)	1.000
1239884495	5	serpin B5 isoform X2 [Canis lupus familiaris]	0.651 (0.42)	1.167 (0.182)	0.200
1239885884	7	protein LEG1 homolog [Canis lupus familiaris]	0.445 (0.154)	0.798 (0.396)	0.556
1239887368	2	myosin-14 isoform X2 [Canis lupus familiaris]	0.792 (0.556)	1.332 (0)	1.000
1239887370	2	myosin-14 isoform X3 [Canis lupus familiaris]	0.792 (0.556)	1.332 (0)	1.000
1239887372	2	myosin-14 isoform X4 [Canis lupus familiaris]	0.792 (0.556)	1.332 (0)	1.000
1239888116	3	LOW QUALITY PROTEIN: CD177 antigen [Canis lupus familiaris]	0.443 (0.544)	1.054 (0.316)	0.095
1239889921	2	ubiquitin-like [Canis lupus familiaris]	0.565 (0.377)	0.823 (0.181)	0.222
1239891563	4	double-headed protease inhibitor, submandibular gland [Canis lupus familiaris]	0.698 (0.092)	0.854 (0.206)	0.029
1239893342	2	F-actin-capping protein subunit beta isoform X3 [Canis lupus familiaris]	0.501 (0)	0.925 (0.168)	0.667
1239893345	2	F-actin-capping protein subunit beta isoform X5 [Canis lupus familiaris]	0.501 (0)	0.925 (0.168)	0.667
1239893349	2	F-actin-capping protein subunit beta isoform X7 [Canis lupus familiaris]	0.501 (0)	0.925 (0.168)	0.667
1239893625	2	calmodulin, partial [Canis lupus familiaris]	0.386 (0.194)	1.41 (0.336)	0.100

1239894395	2	calpastatin isoform X7 [Canis lupus familiaris]	0.684 (0.651)	0.898 (0.742)	0.629
1239894397	2	calpastatin isoform X8 [Canis lupus familiaris]	0.684 (0.651)	0.898 (0.742)	0.629
1239894403	2	calpastatin isoform X13 [Canis lupus familiaris]	0.684 (0.651)	0.898 (0.742)	0.629
1239894411	2	calpastatin isoform X20 [Canis lupus familiaris]	0.684 (0.651)	0.898 (0.742)	0.629
1239898218	2	adipogenesis regulatory factor [Canis lupus familiaris]	1.049 (0.049)	1.196 (0.358)	0.700
1239901610	3	profilin-1 [Canis lupus familiaris]	1.028 (0.377)	1.057 (0.278)	0.421
1239902229	3	polyubiquitin-B [Canis lupus familiaris]	0.568 (0.384)	0.791 (0.201)	0.310
1239905645	4	14-3-3 protein gamma [Canis lupus familiaris]	0.906 (0.146)	0.972 (0.465)	0.690
1239908410	3	glutathione S-transferase Mu 3 isoform X1 [Canis lupus familiaris]	0.895 (0.314)	1.045 (0.166)	0.629
1239908416	10	glutathione S-transferase Mu 1 isoform X2 [Canis lupus familiaris]	0.925 (0.068)	1.02 (0.045)	0.114
1239908722	2	dihydropyrimidine dehydrogenase [NADP(+)] isoform X2 [Canis lupus familiaris]	0.52 (0.237)	0.844 (0.19)	0.400
1239911065	3	peroxiredoxin-6 [Canis lupus familiaris]	1.242 (0.241)	1.173 (0.015)	0.730
1239912330	3	protein S100-A6 [Canis lupus familiaris]	0.419 (0.408)	1.002 (0.404)	0.095
1239913623	6	alpha-1-antitrypsin isoform X1 [Canis lupus familiaris]	1.07 (1.216)	0.723 (0.536)	0.548
1239915208	5	alpha-actinin-1 isoform X2 [Canis lupus familiaris]	0.809 (0.361)	1.001 (0.08)	0.095
1239919186	20	lactoperoxidase [Canis lupus familiaris]	1.069 (1.902)	0.764 (0.03)	0.222
1239922056	3	myosin light polypeptide 6 isoform X3 [Canis lupus familiaris]	0.466 (0.734)	0.96 (0.387)	0.310
1239925350	5	heat shock 70 kDa protein 4 isoform X1 [Canis lupus familiaris]	0.742 (0.445)	1.061 (0.287)	0.095
1239925762	7	gelsolin [Canis lupus familiaris]	1.14 (1.1)	2.12 (1.092)	0.421
1239927670	2	transitional endoplasmic reticulum ATPase isoform X2 [Canis lupus familiaris]	0.762 (0.259)	1.162 (0.127)	0.111
1239928271	4	thioredoxin-like isoform X1 [Canis lupus familiaris]	0.962 (0.371)	0.806 (0.048)	1.000
1239928273	4	thioredoxin-like isoform X2 [Canis lupus familiaris]	0.962 (0.371)	0.806 (0.048)	1.000
1239928570	6	complement C4-A [Canis lupus familiaris]	0.89 (0.228)	0.869 (0.194)	0.629
1239929902	6	cysteine-rich secretory protein 2 isoform X2 [Canis lupus familiaris]	1.093 (0.57)	1.085 (0.254)	0.841
1239929974	3	glutathione S-transferase A4-like [Canis lupus familiaris]	0.43 (0.19)	1.082 (0.258)	0.032
1239931268	2	proteoglycan 4-like [Canis lupus familiaris]	0.678 (0.481)	0.89 (0.08)	0.730
1239933313	6	submaxillary gland androgen-regulated protein 3A isoform X1 [Canis lupus familiaris]	0.626 (0.353)	1.037 (0.581)	0.310
1239933320	2	mucin-7 [Canis lupus familiaris]	0.929 (0.786)	0.753 (0.207)	0.841
1239938912	2	maltase-glucoamylase, intestinal isoform X1 [Canis lupus familiaris]	0.406 (0)	1.22 (0.524)	0.667
1239945861	2	mucin-5B isoform X1 [Canis lupus familiaris]	0.691 (0.378)	1.028 (0.306)	0.400
1239950142	7	hyaluronidase-1 isoform X3 [Canis lupus familiaris]	0.812 (0.444)	0.778 (0.16)	1.000

1239951047	2	LOW QUALITY PROTEIN: caspase-14 [Canis lupus familiaris]	0.578 (0.409)	1.366 (0.428)	0.114
1239951704	28	complement C3 [Canis lupus familiaris]	0.467 (0.432)	0.975 (0.104)	0.151
1239953679	2	folate receptor beta isoform X1 [Canis lupus familiaris]	0.39 (0)	1.207 (0.605)	0.667
1239955263	3	malate dehydrogenase, mitochondrial-like [Canis lupus familiaris]	0.447 (0.139)	0.88 (0.281)	0.008
1239955652	3	L-lactate dehydrogenase A chain-like isoform X1 [Canis lupus familiaris]	0.691 (0.385)	0.919 (0.336)	1.000
1239955970	17	plastin-2 isoform X1 [Canis lupus familiaris]	0.538 (0.622)	1.111 (0.161)	0.016
1239956251	2	olfactomedin-4 [Canis lupus familiaris]	1.489 (0.604)	0.842 (0.148)	0.190
1239957836	3	ceruloplasmin isoform X2 [Canis lupus familiaris]	0.464 (0.397)	1.028 (0.074)	0.111
1239957839	3	ceruloplasmin isoform X4 [Canis lupus familiaris]	0.464 (0.397)	1.028 (0.074)	0.111
1239958986	5	LOW QUALITY PROTEIN: L-lactate dehydrogenase A chain-like [Canis lupus familiaris]	0.481 (0.61)	0.853 (0.379)	0.421
1239959951	2	vomeromodulin-like [Canis lupus familiaris]	0.968 (0.228)	1.083 (0.302)	0.667
1239961995	2	phosphatidylethanolamine-binding protein 4 isoform X2 [Canis lupus familiaris]	0.924 (0.116)	1.051 (0.328)	0.700
1239961997	2	phosphatidylethanolamine-binding protein 4 isoform X3 [Canis lupus familiaris]	0.956 (0.568)	1.1 (0.328)	1.000
1239961999	3	phosphatidylethanolamine-binding protein 4 isoform X4 [Canis lupus familiaris]	0.924 (0.112)	1.051 (0.284)	0.700
1239962001	3	phosphatidylethanolamine-binding protein 4 isoform X5 [Canis lupus familiaris]	0.924 (0.112)	1.051 (0.284)	0.700
1239963916	2	glyceraldehyde-3-phosphate dehydrogenase-like [Canis lupus familiaris]	1.058 (0.467)	0.824 (0.004)	0.016
1239964305	2	actin-related protein 2/3 complex subunit 3 isoform X1 [Canis lupus familiaris]	0.364 (0.615)	0.851 (0.31)	0.222
1239965760	3	keratin, type II cytoskeletal 2 oral isoform X1 [Canis lupus familiaris]	0.329 (0.08)	0.465 (1.687)	0.700
1239967247	5	pregnancy zone protein-like isoform X3 [Canis lupus familiaris]	0.838 (0.365)	1.235 (0.104)	0.086
1239967618	6	alpha-2-macroglobulin-like protein 1 isoform X1 [Canis lupus familiaris]	0.73 (0)	1.154 (0.14)	0.667
1239967622	6	alpha-2-macroglobulin-like protein 1 isoform X2 [Canis lupus familiaris]	0.73 (0)	1.154 (0.14)	0.667
1239968164	7	mucin-19 [Canis lupus familiaris]	0.61 (0.219)	0.907 (0.322)	0.343
1239968362	2	poly(U)-specific endoribonuclease [Canis lupus familiaris]	1.031 (0.44)	0.871 (0.176)	1.000
1239971228	3	fatty acid-binding protein, epidermal isoform X1 [Canis lupus familiaris]	0.792 (0.142)	1.047 (0.249)	0.310
1239971230	3	fatty acid-binding protein, epidermal isoform X2 [Canis lupus familiaris]	0.792 (0.142)	1.047 (0.249)	0.310
1239971232	3	fatty acid-binding protein, epidermal isoform X3 [Canis lupus familiaris]	0.792 (0.142)	1.047 (0.249)	0.310
1239972768	7	pyruvate kinase PKM isoform X1 [Canis lupus familiaris]	1.07 (0.305)	0.832 (0.053)	0.413
1239976879	3	mucin-4 [Canis lupus familiaris]	0.562 (0)	0.858 (0.122)	0.667
1239979297	6	leukocyte elastase inhibitor [Canis lupus familiaris]	0.624 (0.597)	0.862 (0.279)	1.000
1239979503	3	LOW QUALITY PROTEIN: uncharacterized protein LOC106558262 [Canis lupus familiaris]	0.407 (0.41)	0.741 (0.166)	0.310
1239979741	2	histone H2A type 1-E [Canis lupus familiaris]	0.575 (0.406)	0.821 (0.058)	0.690

1239979771	2	histone H2A type 1 [Canis lupus familiaris]	0.575 (0.406)	0.821 (0.058)	0.690
1239979774	2	histone H2A type 1-H [Canis lupus familiaris]	0.575 (0.406)	0.821 (0.058)	0.690
1239981819	4	actin-related protein 2/3 complex subunit 2 [Canis lupus familiaris]	0.575 (0.329)	0.86 (0.209)	0.100
1239982239	4	fibronectin isoform X2 [Canis lupus familiaris]	0.785 (0.703)	1.041 (0.501)	0.629
1239982241	4	fibronectin isoform X3 [Canis lupus familiaris]	0.785 (0.703)	1.041 (0.501)	0.629
1239982243	4	fibronectin isoform X6 [Canis lupus familiaris]	0.785 (0.703)	1.041 (0.501)	0.629
1239982245	4	fibronectin isoform X8 [Canis lupus familiaris]	0.785 (0.703)	1.041 (0.501)	0.629
1239983604	12	plastin-3 [Canis lupus familiaris]	0.839 (0.258)	0.991 (0.243)	0.548
1239986810	3	fatty acid-binding protein, epidermal [Canis lupus familiaris]	0.792 (0.142)	1.047 (0.249)	0.310
1258500452	5	Chain B, Endoplasmin	0.722 (0.594)	0.926 (0.44)	0.629
1335512822	2	Chain A, Endoplasmin	1.395 (0.83)	1.069 (0)	1.000
1340189905	2	immunoglobulin heavy chain variable region, partial [Canis lupus familiaris]	1.222 (2.119)	0.88 (0.376)	0.556
1340239380	2	immunoglobulin heavy chain variable region, partial [Canis lupus familiaris]	1.594 (0.734)	1.198 (0.55)	0.421
1374502923	10	Chain D, Lipocalin-Can f 6 allergen	0.726 (0.49)	0.778 (0.402)	1.000
1379069777	2	Chain B, Endoplasmin	1.395 (0.83)	1.069 (0)	1.000
1391723726	3	protein S100-A4 isoform 1 [Canis lupus familiaris]	0.258 (0.607)	0.87 (0.304)	0.095
1418192575	7	submaxillary mucin-like protein, partial [Canis lupus dingo]	0.599 (0.306)	1.089 (0.45)	0.200
1418194692	6	angiopoietin-related protein 5-like [Canis lupus dingo]	1.65 (1.354)	1.164 (0.422)	0.421
1418195836	21	myosin-9 [Canis lupus dingo]	0.522 (0.449)	1.1 (0.424)	0.151
1418197013	2	calmodulin-2 [Canis lupus dingo]	0.88 (0.298)	1.341 (0.488)	0.008
1418200086	3	mucin-4 [Canis lupus dingo]	0.562 (0)	0.858 (0.122)	0.667
1418202110	4	actin-related protein 3 [Canis lupus dingo]	0.515 (0.46)	1.048 (0.597)	0.151
1418202784	4	peptidyl-prolyl cis-trans isomerase A isoform X1 [Canis lupus dingo]	1.247 (0.087)	0.947 (0.641)	0.286
1418202786	2	peptidyl-prolyl cis-trans isomerase A isoform X2 [Canis lupus dingo]	0.92 (0.167)	1.091 (0.358)	0.413
1418204456	2	histone H2A type 1-E [Canis lupus dingo]	0.575 (0.406)	0.821 (0.058)	0.690
1418204540	2	histone H2A type 1-H-like [Canis lupus dingo]	0.575 (0.406)	0.821 (0.058)	0.690
1418204605	3	proteasome subunit alpha type-1 [Canis lupus dingo]	0.592 (0)	0.968 (0.236)	0.667
1418205354	3	ribosyldihydronicotinamide dehydrogenase [quinone] [Canis lupus dingo]	0.962 (0.356)	0.879 (0.1)	1.000
1418205376	11	leukocyte elastase inhibitor [Canis lupus dingo]	0.628 (0.307)	0.88 (0.062)	0.190
1418206782	2	LOW QUALITY PROTEIN: mucin-5B [Canis lupus dingo]	0.619 (0.263)	1.181 (0.383)	0.056
1418207290	2	60S acidic ribosomal protein P2 [Canis lupus dingo]	0.895 (0)	1.036 (0.368)	1.000

1418208278	2	prothymosin alpha [Canis lupus dingo]	0.432 (0.155)	1.056 (0.286)	0.032
1418211040	6	macrophage-capping protein isoform X1 [Canis lupus dingo]	0.63 (0.355)	0.993 (0.16)	0.222
1418211046	4	macrophage-capping protein isoform X2 [Canis lupus dingo]	0.514 (0.407)	1.03 (0.294)	0.095
1418212080	7	xanthine dehydrogenase/oxidase isoform X1 [Canis lupus dingo]	0.786 (0.422)	1.066 (0.232)	0.222
1418212082	7	xanthine dehydrogenase/oxidase isoform X2 [Canis lupus dingo]	0.786 (0.422)	1.066 (0.232)	0.222
1418213116	3	14-3-3 protein theta [Canis lupus dingo]	0.785 (0.286)	1.191 (0.193)	0.032
1418213478	2	protein S100-A11, partial [Canis lupus dingo]	0.396 (0.246)	1.136 (0.528)	0.032
1418213540	2	cornifin-A [Canis lupus dingo]	0.591 (0.214)	1.168 (0.562)	0.056
1418213542	5	involucrin [Canis lupus dingo]	1.002 (0.347)	0.853 (0.31)	1.000
1418213935	6	alpha-2-macroglobulin-like protein 1 isoform X1 [Canis lupus dingo]	0.73 (0)	1.154 (0.14)	0.667
1418213939	6	alpha-2-macroglobulin-like protein 1 isoform X2 [Canis lupus dingo]	0.73 (0)	1.154 (0.14)	0.667
1418213974	3	alpha-2-macroglobulin [Canis lupus dingo]	0.457 (0.77)	1.049 (0.212)	0.548
1418213976	6	pregnancy zone protein-like isoform X1 [Canis lupus dingo]	0.826 (0.409)	1.155 (0.044)	0.229
1418213978	6	pregnancy zone protein-like isoform X2 [Canis lupus dingo]	0.826 (0.409)	1.155 (0.044)	0.229
1418213980	5	pregnancy zone protein-like isoform X3 [Canis lupus dingo]	0.838 (0.365)	1.235 (0.104)	0.086
1418214537	2	ovostatin homolog 2-like [Canis lupus dingo]	0.724 (0.294)	0.866 (0.21)	1.000
1418215404	6	cathelicidin antimicrobial peptide [Canis lupus dingo]	0.68 (0.289)	1.106 (0.636)	0.310
1418215838	9	hyaluronidase-1 isoform X2 [Canis lupus dingo]	0.886 (0.983)	0.692 (0.136)	0.222
1418216286	25	transketolase isoform X1 [Canis lupus dingo]	0.648 (0.397)	1.061 (0.086)	0.032
1418216288	25	transketolase isoform X2 [Canis lupus dingo]	0.648 (0.397)	1.061 (0.086)	0.032
1418218016	4	leucine-rich alpha-2-glycoprotein [Canis lupus dingo]	1.02 (0.387)	0.86 (0.174)	0.857
1418218430	30	complement C3 [Canis lupus dingo]	0.484 (0.449)	0.988 (0.112)	0.151
1418219369	6	calreticulin [Canis lupus dingo]	0.709 (0.035)	0.63 (0.014)	0.057
1418220429	33	lactotransferrin [Canis lupus dingo]	0.919 (0.384)	0.895 (0.076)	0.667
1418220639	4	neutrophil elastase [Canis lupus dingo]	0.444 (0.363)	0.541 (0.346)	0.548
1418221457	9	keratin, type II cytoskeletal 4 [Canis lupus dingo]	0.244 (0.112)	0.372 (0.095)	0.079
1418221463	4	keratin, type II cytoskeletal 1 [Canis lupus dingo]	0.419 (0.084)	0.562 (0.19)	0.063
1418221469	4	keratin, type II cytoskeletal 5 [Canis lupus dingo]	0.253 (0.153)	0.43 (0.077)	0.190
1418221475	6	keratin, type II cytoskeletal 6A-like [Canis lupus dingo]	0.513 (0.174)	0.838 (1.171)	0.629
1418221668	3	keratin, type II cytoskeletal 2 oral-like [Canis lupus dingo]	0.329 (0.08)	0.465 (1.687)	0.700
1418222123	2	folate receptor beta isoform X1 [Canis lupus dingo]	0.39 (0)	1.207 (0.605)	0.667

1418222127	2	folate receptor beta isoform X2 [Canis lupus dingo]	0.39 (0)	1.207 (0.605)	0.667
1418222274	13	hemoglobin subunit beta [Canis lupus dingo]	0.491 (0.262)	0.5 (0.088)	1.000
1418222276	7	hemoglobin subunit beta-like [Canis lupus dingo]	0.53 (0.34)	0.487 (0.102)	1.000
1418222280	2	hemoglobin subunit epsilon [Canis lupus dingo]	1.716 (0.372)	1.191 (0.411)	0.667
1418222965	3	proteasome subunit alpha type-1 [Canis lupus dingo]	0.592 (0)	0.968 (0.236)	0.667
1418223224	10	L-lactate dehydrogenase A chain isoform X1 [Canis lupus dingo]	0.634 (0.51)	0.899 (0.26)	0.310
1418225081	7	glucose-6-phosphate 1-dehydrogenase isoform X1 [Canis lupus dingo]	0.849 (0.519)	0.74 (0.198)	1.000
1418225083	7	glucose-6-phosphate 1-dehydrogenase isoform X2 [Canis lupus dingo]	0.849 (0.519)	0.74 (0.198)	1.000
1418225135	6	filamin-A isoform X1 [Canis lupus dingo]	0.464 (0.437)	1.163 (0.117)	0.032
1418225137	6	filamin-A isoform X2 [Canis lupus dingo]	0.464 (0.437)	1.163 (0.117)	0.032
1418227068	2	acetyl-coenzyme A synthetase 2-like, mitochondrial [Canis lupus dingo]	0.613 (0.155)	1.522 (0.125)	0.057
1418228196	2	trefoil factor 3 [Canis lupus dingo]	1.047 (0.244)	0.876 (0.176)	0.556
1418230422	3	phosphatidylethanolamine-binding protein 4 isoform X2 [Canis lupus dingo]	0.924 (0.116)	1.051 (0.328)	0.700
1418230424	2	phosphatidylethanolamine-binding protein 4 isoform X3 [Canis lupus dingo]	0.956 (0.568)	1.1 (0.328)	1.000
1418230426	4	phosphatidylethanolamine-binding protein 4 isoform X4 [Canis lupus dingo]	0.924 (0.112)	1.051 (0.284)	0.700
1418230428	4	phosphatidylethanolamine-binding protein 4 isoform X5 [Canis lupus dingo]	0.924 (0.112)	1.051 (0.284)	0.700
1418230430	4	phosphatidylethanolamine-binding protein 4 isoform X6 [Canis lupus dingo]	0.924 (0.112)	1.051 (0.284)	0.700
1418230432	4	phosphatidylethanolamine-binding protein 4 isoform X1 [Canis lupus dingo]	0.924 (0.112)	1.051 (0.284)	0.700
1418231166	2	prothymosin alpha [Canis lupus dingo]	0.432 (0.155)	1.056 (0.286)	0.032
1418240337	6	elongation factor 2 [Canis lupus dingo]	0.524 (0.288)	0.923 (0.104)	0.200
1418241889	15	apolipoprotein A-I [Canis lupus dingo]	0.38 (0.192)	0.561 (0.303)	0.730
1418243364	6	moesin isoform X2 [Canis lupus dingo]	1.138 (0.648)	0.965 (0.011)	1.000
1418243839	2	glyceraldehyde-3-phosphate dehydrogenase-like isoform X1 [Canis lupus dingo]	1.058 (0.467)	0.824 (0.004)	0.016
1418243841	2	glyceraldehyde-3-phosphate dehydrogenase-like isoform X2 [Canis lupus dingo]	1.058 (0.467)	0.824 (0.004)	0.016
1418245560	3	glutathione S-transferase omega-1 [Canis lupus dingo]	0.974 (0.122)	1.28 (0)	0.667
1418246878	11	LOW QUALITY PROTEIN: deleted in malignant brain tumors 1 protein [Canis lupus dingo]	1.125 (0.377)	0.925 (0.17)	0.151
1418248118	4	actin, cytoplasmic 2 [Canis lupus dingo]	1.145 (0.154)	0.76 (0.14)	0.190
1418249227	2	ubiquitin-like modifier-activating enzyme 1 [Canis lupus dingo]	0.781 (0.318)	1.041 (0)	1.000
1418249549	11	BPI fold-containing family B member 2 [Canis lupus dingo]	1.044 (0.276)	0.805 (0.078)	0.286
1418249553	16	BPI fold-containing family A member 2 [Canis lupus dingo]	0.479 (1.16)	0.508 (0.383)	1.000
1418249573	2	antileukoproteinase-like [Canis lupus dingo]	0.803 (0.313)	0.937 (0.534)	0.629

1418250613	11	protein-glutamine gamma-glutamyltransferase E [Canis lupus dingo]	0.841 (0.264)	0.92 (0.142)	0.841
1418251074	13	BPI fold-containing family B member 1 [Canis lupus dingo]	0.568 (1.444)	0.476 (0.206)	0.841
1418252030	3	14-3-3 protein beta/alpha [Canis lupus dingo]	1.216 (1.271)	1.021 (0.539)	0.548
1418252189	7	matrix metalloproteinase-9 [Canis lupus dingo]	0.694 (0.372)	0.896 (0.432)	0.690
1418253446	2	histone H2A type 1-B/E [Canis lupus dingo]	0.575 (0.406)	0.821 (0.058)	0.690
1418253448	2	histone H2A type 1-E-like [Canis lupus dingo]	0.575 (0.406)	0.821 (0.058)	0.690
1418253483	2	histone H2A type 1-E-like [Canis lupus dingo]	0.575 (0.406)	0.821 (0.058)	0.690
1418253487	3	uncharacterized protein LOC112674397 isoform X2 [Canis lupus dingo]	0.407 (0.41)	0.741 (0.166)	0.310
1418253519	3	LOW QUALITY PROTEIN: uncharacterized protein LOC112674420 [Canis lupus dingo]	0.407 (0.41)	0.741 (0.166)	0.310
1418253529	3	histone H4-like [Canis lupus dingo]	0.407 (0.41)	0.741 (0.166)	0.310
1418253569	3	uncharacterized protein LOC112674463 [Canis lupus dingo]	0.407 (0.41)	0.741 (0.166)	0.310
1418256156	2	gamma-enolase [Canis lupus dingo]	1.661 (1.274)	1.121 (0.335)	0.032
1418257040	6	pyruvate kinase PKM isoform X2 [Canis lupus dingo]	1.026 (0.328)	0.822 (0.018)	0.730
1418257042	7	pyruvate kinase PKM isoform X3 [Canis lupus dingo]	1.07 (0.305)	0.832 (0.053)	0.413
1418257044	6	pyruvate kinase PKM isoform X4 [Canis lupus dingo]	1.07 (0.409)	0.826 (0.062)	0.413
1418258110	3	annexin A2 [Canis lupus dingo]	0.408 (0.31)	0.884 (0.551)	0.200
1418258143	2	proteasome subunit alpha type-1-like [Canis lupus dingo]	0.473 (0)	0.944 (0.175)	0.667
1418259105	4	protein disulfide-isomerase A3 [Canis lupus dingo]	0.765 (0.043)	1.072 (0.401)	0.421
1418260227	2	uncharacterized protein LOC112676265 [Canis lupus dingo]	2.449 (1.228)	0.828 (0.255)	0.400
1418260233	2	glyceraldehyde-3-phosphate dehydrogenase-like [Canis lupus dingo]	1.058 (0.467)	0.824 (0.004)	0.016
1418260846	3	polyubiquitin-C [Canis lupus dingo]	0.568 (0.384)	0.791 (0.201)	0.310
1418261548	2	actin-related protein 2/3 complex subunit 3 isoform X2 [Canis lupus dingo]	0.364 (0.615)	0.851 (0.31)	0.222
1418261550	2	actin-related protein 2/3 complex subunit 3 isoform X3 [Canis lupus dingo]	0.364 (0.615)	0.851 (0.31)	0.222
1418264299	13	major allergen Can f 1 [Canis lupus dingo]	0.492 (0.041)	0.88 (0.461)	0.016
1418264760	5	endoplasmic reticulum chaperone BiP [Canis lupus dingo]	0.873 (0.493)	0.9 (0.366)	0.421
1418266307	7	minor allergen Can f 2 [Canis lupus dingo]	1.167 (0.36)	0.83 (0.185)	0.063
1418267412	4	14-3-3 protein epsilon isoform X1 [Canis lupus dingo]	0.598 (0.501)	1.069 (0.461)	0.151
1418267416	3	14-3-3 protein epsilon isoform X3 [Canis lupus dingo]	0.598 (0.464)	1.069 (0.488)	0.151
1418267418	3	14-3-3 protein epsilon isoform X4 [Canis lupus dingo]	0.598 (0.464)	1.069 (0.488)	0.151
1418269617	3	myosin light polypeptide 6 isoform X2 [Canis lupus dingo]	0.466 (0.734)	0.96 (0.387)	0.310
1418270515	2	poly(U)-specific endoribonuclease [Canis lupus dingo]	1.031 (0.44)	0.871 (0.176)	1.000

1418273448	3	L-lactate dehydrogenase B chain-like [Canis lupus dingo]	1.029 (0.308)	0.77 (0.108)	0.114
1418280689	8	fructose-bisphosphate aldolase A [Canis lupus dingo]	0.516 (0.307)	0.925 (0.29)	0.095
1418282931	2	apoptosis-associated speck-like protein containing a CARD isoform X2 [Canis lupus dingo]	1.274 (0.318)	1.73 (0.602)	0.667
1418286550	5	myosin-11 isoform X1 [Canis lupus dingo]	0.774 (0.459)	1.235 (0.724)	0.222
1418286554	5	myosin-11 isoform X3 [Canis lupus dingo]	0.774 (0.459)	1.235 (0.724)	0.222
1418286556	5	myosin-11 isoform X4 [Canis lupus dingo]	0.774 (0.459)	1.235 (0.724)	0.222
1418288349	11	glutathione S-transferase Mu 1 isoform X1 [Canis lupus dingo]	0.899 (0.115)	1.014 (0.032)	0.413
1418288800	2	dihydropyrimidine dehydrogenase [NADP(+)] isoform X1 [Canis lupus dingo]	0.52 (0.237)	0.844 (0.19)	0.400
1418288802	2	dihydropyrimidine dehydrogenase [NADP(+)] isoform X2 [Canis lupus dingo]	0.52 (0.237)	0.844 (0.19)	0.400
1418290456	6	complement C4-A-like [Canis lupus dingo]	0.89 (0.228)	0.869 (0.194)	0.629
1418291961	2	platelet-activating factor acetylhydrolase isoform X1 [Canis lupus dingo]	0.986 (0.574)	0.686 (0.303)	0.114
1418291973	2	platelet-activating factor acetylhydrolase isoform X3 [Canis lupus dingo]	0.986 (0.574)	0.686 (0.303)	0.114
1418292147	2	glutathione S-transferase A2 [Canis lupus dingo]	0.4 (0.681)	0.963 (0.584)	0.629
1418292151	3	glutathione S-transferase A4-like [Canis lupus dingo]	0.43 (0.19)	1.082 (0.258)	0.032
1418292394	2	glyceraldehyde-3-phosphate dehydrogenase isoform X1 [Canis lupus dingo]	1.058 (0.467)	0.824 (0.004)	0.016
1418292396	2	glyceraldehyde-3-phosphate dehydrogenase isoform X2 [Canis lupus dingo]	1.058 (0.467)	0.824 (0.004)	0.016
1418293878	8	adenylyl cyclase-associated protein 1 isoform X1 [Canis lupus dingo]	0.459 (0.386)	0.987 (0.265)	0.095
1418293884	8	adenylyl cyclase-associated protein 1 isoform X2 [Canis lupus dingo]	0.459 (0.386)	0.987 (0.265)	0.095
1418294943	4	peroxiredoxin-1 [Canis lupus dingo]	0.599 (0.093)	0.961 (0.385)	0.222
1418297237	3	alpha-1B-glycoprotein [Canis lupus dingo]	0.932 (0.541)	0.498 (0.038)	0.016
1418298925	2	serpin B10 [Canis lupus dingo]	1.088 (0.024)	0.721 (0.164)	0.100
1418298970	5	serpin B5 isoform X1 [Canis lupus dingo]	0.651 (0.42)	1.167 (0.182)	0.200
1418298974	5	serpin B5 isoform X2 [Canis lupus dingo]	0.651 (0.42)	1.167 (0.182)	0.200
1418299581	4	L-lactate dehydrogenase B chain-like [Canis lupus dingo]	0.914 (0.102)	0.77 (0.094)	0.114
1418300273	7	ezrin isoform X2 [Canis lupus dingo]	0.795 (0.101)	0.983 (0.244)	0.310
1418301038	7	protein LEG1 homolog [Canis lupus dingo]	0.445 (0.154)	0.798 (0.396)	0.556
1418301814	4	guanine deaminase [Canis lupus dingo]	0.581 (0.837)	0.977 (0.248)	0.690
1418303494	9	kallikrein-1 isoform X1 [Canis lupus dingo]	1.934 (0.664)	0.859 (0.076)	0.190
1418303496	9	kallikrein-1 isoform X2 [Canis lupus dingo]	1.934 (0.664)	0.859 (0.076)	0.190
1418303522	3	kallikrein-1-like isoform X1 [Canis lupus dingo]	1.599 (0.094)	0.753 (0.194)	0.029
1418303524	3	kallikrein-1-like isoform X2 [Canis lupus dingo]	1.599 (0.094)	0.753 (0.194)	0.029

1418303612	2	myosin-14 isoform X1 [Canis lupus dingo]	0.792 (0.556)	1.332 (0)	1.000
1418303616	2	myosin-14 isoform X2 [Canis lupus dingo]	0.792 (0.556)	1.332 (0)	1.000
1418303618	2	myosin-14 isoform X3 [Canis lupus dingo]	0.792 (0.556)	1.332 (0)	1.000
1418303620	2	myosin-14 isoform X4 [Canis lupus dingo]	0.792 (0.556)	1.332 (0)	1.000
1418304395	2	calmodulin-3 isoform X2 [Canis lupus dingo]	0.788 (0.199)	1.088 (0.054)	0.016
1418304784	3	CD177 antigen [Canis lupus dingo]	0.443 (0.544)	1.054 (0.316)	0.095
1418304786	4	ly6/PLAUR domain-containing protein 3 [Canis lupus dingo]	0.669 (0.117)	0.891 (0.301)	0.095
1418305257	2	IgGFc-binding protein isoform X1 [Canis lupus dingo]	1.378 (2.409)	1.024 (0.261)	0.310
1418305259	2	IgGFc-binding protein isoform X2 [Canis lupus dingo]	1.32 (2.233)	1.015 (0.278)	0.310
1418305407	13	alpha-actinin-4 isoform X1 [Canis lupus dingo]	0.612 (0.382)	1.066 (0.033)	0.056
1418305409	12	alpha-actinin-4 isoform X2 [Canis lupus dingo]	0.612 (0.384)	1.066 (0.041)	0.056
1418305411	13	alpha-actinin-4 isoform X3 [Canis lupus dingo]	0.612 (0.382)	1.066 (0.033)	0.056
1418305415	11	alpha-actinin-4 isoform X5 [Canis lupus dingo]	0.612 (0.361)	1.075 (0.041)	0.095
1418305580	15	LOW QUALITY PROTEIN: glucose-6-phosphate isomerase [Canis lupus dingo]	0.995 (0.063)	1.056 (0.182)	0.794
1418307145	4	malate dehydrogenase, mitochondrial [Canis lupus dingo]	0.488 (0.139)	0.881 (0.205)	0.008
1418307159	3	heat shock protein beta-1 [Canis lupus dingo]	1.207 (0.52)	1.195 (0.257)	1.000
1418307161	4	14-3-3 protein gamma [Canis lupus dingo]	0.906 (0.146)	0.972 (0.465)	0.690
1418308718	3	cadherin-1 [Canis lupus dingo]	0.497 (0.044)	0.872 (0.35)	0.100
1418308783	9	NAD(P)H dehydrogenase [quinone] 1 [Canis lupus dingo]	0.632 (0.338)	0.838 (0.396)	0.548
1418309542	3	coactosin-like protein [Canis lupus dingo]	0.873 (0.817)	1.168 (0.391)	0.690
1418310052	17	alpha-enolase isoform X1 [Canis lupus dingo]	0.6 (0.255)	1.03 (0.03)	0.190
1418310054	15	alpha-enolase isoform X2 [Canis lupus dingo]	0.565 (0.241)	0.981 (0.038)	0.190
1418311328	3	ubiquitin [Canis lupus dingo]	0.568 (0.384)	0.791 (0.201)	0.310
1418312167	3	polyubiquitin-B [Canis lupus dingo]	0.568 (0.384)	0.791 (0.201)	0.310
1418312500	4	myosin-10 isoform X2 [Canis lupus dingo]	1.162 (0.713)	1.036 (0.399)	0.794
1418312506	4	myosin-10 isoform X5 [Canis lupus dingo]	1.162 (0.713)	1.036 (0.399)	0.794
1418312512	4	myosin-10 isoform X6 [Canis lupus dingo]	1.162 (0.713)	1.036 (0.399)	0.794
1418313130	5	profilin-1 [Canis lupus dingo]	1.002 (0.17)	1.001 (0.256)	0.690
1418313159	4	beta-enolase isoform X2 [Canis lupus dingo]	0.716 (0.569)	1.285 (0.668)	0.310
1418313468	2	tropomyosin alpha-3 chain isoform X7 [Canis lupus dingo]	0.348 (0.873)	0.98 (0.143)	0.548
1418313584	5	protein S100-A2 [Canis lupus dingo]	0.541 (0.151)	0.962 (0.03)	0.016

1418313614	8	protein S100-A12-like [Canis lupus dingo]	0.268 (0.526)	1.199 (0.45)	0.032
1418314253	3	desmoglein-1 [Canis lupus dingo]	0.794 (0.245)	1.155 (0.28)	0.229
1418314263	6	desmocollin-2 isoform X2 [Canis lupus dingo]	0.933 (0.208)	0.96 (0.289)	0.841
1418314267	6	desmocollin-2 isoform X4 [Canis lupus dingo]	0.933 (0.208)	0.96 (0.289)	0.841
1418314603	2	myosin regulatory light polypeptide 9 isoform X1 [Canis lupus dingo]	0.488 (0.483)	0.887 (0.149)	0.310
1418314796	2	elongation factor 1-gamma-like [Canis lupus dingo]	0.76 (0.249)	0.895 (0.316)	0.310
1418315363	5	cofilin-1-like [Canis lupus dingo]	0.887 (0.541)	0.956 (0.136)	0.841
1418316200	2	actin-related protein 2/3 complex subunit 5 [Canis lupus dingo]	0.407 (0.291)	1.092 (0.24)	0.200
1418316704	3	peroxiredoxin-6 [Canis lupus dingo]	1.242 (0.241)	1.173 (0.015)	0.730
1418318928	12	plastin-3 isoform X1 [Canis lupus dingo]	0.839 (0.258)	0.991 (0.243)	0.548
1418319535	3	L-lactate dehydrogenase B chain-like [Canis lupus dingo]	1.029 (0.308)	0.77 (0.108)	0.114
1418320363	2	transitional endoplasmic reticulum ATPase [Canis lupus dingo]	0.762 (0.259)	1.162 (0.127)	0.111
1418320657	10	allergen Fel d 4-like [Canis lupus dingo]	0.726 (0.49)	0.778 (0.402)	1.000
1418321329	6	prostaglandin reductase 1 [Canis lupus dingo]	0.521 (0.506)	0.988 (0.26)	0.421
1418321566	4	thioredoxin [Canis lupus dingo]	0.962 (0.371)	0.806 (0.048)	1.000
1418322411	7	gelsolin isoform X1 [Canis lupus dingo]	1.14 (1.1)	2.12 (1.092)	0.421
1418322413	7	gelsolin isoform X2 [Canis lupus dingo]	1.14 (1.1)	2.12 (1.092)	0.421
1418322415	7	gelsolin isoform X3 [Canis lupus dingo]	1.14 (1.1)	2.12 (1.092)	0.421
1418322757	5	heat shock 70 kDa protein 4 isoform X3 [Canis lupus dingo]	0.742 (0.445)	1.061 (0.287)	0.095
1418322788	2	alpha-1-acid glycoprotein 1-like [Canis lupus dingo]	0.337 (0.228)	0.928 (0.63)	0.200
1418323952	3	fatty acid-binding protein 5 [Canis lupus dingo]	0.792 (0.142)	1.047 (0.249)	0.310
1418324508	2	maltase-glucoamylase, intestinal isoform X1 [Canis lupus dingo]	0.406 (0)	1.22 (0.524)	0.667
1418324514	2	maltase-glucoamylase, intestinal isoform X2 [Canis lupus dingo]	0.406 (0)	1.22 (0.524)	0.667
1418325065	8	prosaposin isoform X6 [Canis lupus dingo]	0.864 (0.188)	0.921 (0.3)	0.421
1418326635	5	vinculin isoform X1 [Canis lupus dingo]	0.338 (0.266)	1.211 (0.344)	0.200
1418326637	5	vinculin isoform X2 [Canis lupus dingo]	0.338 (0.266)	1.211 (0.344)	0.200
1418327082	3	L-lactate dehydrogenase B chain-like [Canis lupus dingo]	0.914 (0.102)	0.77 (0.094)	0.114
1418328391	8	opiorphin prepropeptide [Canis lupus dingo]	0.602 (0.364)	1.006 (0.503)	0.310
1418328547	2	LOW QUALITY PROTEIN: serum albumin-like [Canis lupus dingo]	0.585 (0.594)	1.089 (0.331)	0.841
1418328657	8	vitamin D-binding protein [Canis lupus dingo]	0.673 (0.735)	1.036 (0.157)	0.548
1418329836	2	succinyl-CoA:3-ketoacid coenzyme A transferase 1, mitochondrial [Canis lupus dingo]	0.468 (0.196)	0.981 (0.005)	0.333

1418331169	3	ras GTPase-activating-like protein IQGAP1 [Canis lupus dingo]	1.048 (0.323)	1.107 (0.646)	0.629
1418331380	2	calpastatin isoform X1 [Canis lupus dingo]	0.684 (0.651)	0.898 (0.742)	0.629
1418331382	2	calpastatin isoform X2 [Canis lupus dingo]	0.684 (0.651)	0.898 (0.742)	0.629
1418331384	2	calpastatin isoform X3 [Canis lupus dingo]	0.684 (0.651)	0.898 (0.742)	0.629
1418331386	2	calpastatin isoform X4 [Canis lupus dingo]	0.684 (0.651)	0.898 (0.742)	0.629
1418331388	2	calpastatin isoform X5 [Canis lupus dingo]	0.684 (0.651)	0.898 (0.742)	0.629
1418331390	2	calpastatin isoform X6 [Canis lupus dingo]	0.684 (0.651)	0.898 (0.742)	0.629
1418331392	2	calpastatin isoform X7 [Canis lupus dingo]	0.684 (0.651)	0.898 (0.742)	0.629
1418331394	2	calpastatin isoform X8 [Canis lupus dingo]	0.684 (0.651)	0.898 (0.742)	0.629
1418331396	2	calpastatin isoform X9 [Canis lupus dingo]	0.684 (0.651)	0.898 (0.742)	0.629
1418331398	2	calpastatin isoform X10 [Canis lupus dingo]	0.684 (0.651)	0.898 (0.742)	0.629
1418331400	2	calpastatin isoform X11 [Canis lupus dingo]	0.684 (0.651)	0.898 (0.742)	0.629
1418331402	2	calpastatin isoform X12 [Canis lupus dingo]	0.684 (0.651)	0.898 (0.742)	0.629
1418331404	2	calpastatin isoform X13 [Canis lupus dingo]	0.684 (0.651)	0.898 (0.742)	0.629
1418331406	2	calpastatin isoform X14 [Canis lupus dingo]	0.684 (0.651)	0.898 (0.742)	0.629
1418331408	2	calpastatin isoform X15 [Canis lupus dingo]	0.684 (0.651)	0.898 (0.742)	0.629
1418331410	2	calpastatin isoform X16 [Canis lupus dingo]	0.684 (0.651)	0.898 (0.742)	0.629
1418331412	2	calpastatin isoform X17 [Canis lupus dingo]	0.684 (0.651)	0.898 (0.742)	0.629
1418331414	2	calpastatin isoform X18 [Canis lupus dingo]	0.684 (0.651)	0.898 (0.742)	0.629
1418331416	2	calpastatin isoform X19 [Canis lupus dingo]	0.684 (0.651)	0.898 (0.742)	0.629
1418333042	2	actin, clone 302-like [Canis lupus dingo]	1.091 (0.143)	0.74 (0.178)	0.190
1418335689	2	malate dehydrogenase, mitochondrial-like [Canis lupus dingo]	0.488 (0.103)	0.881 (0.395)	0.032
1418335936	3	purine nucleoside phosphorylase [Canis lupus dingo]	0.873 (0.227)	1.116 (0.537)	0.413
1418336346	5	fibrinogen alpha chain [Canis lupus dingo]	0.402 (0.399)	0.831 (0.375)	0.222
1418336465	4	fibrinogen beta chain [Canis lupus dingo]	0.749 (0.567)	0.91 (0.446)	0.421
1418337563	6	keratin, type I cytoskeletal 10 isoform X1 [Canis lupus dingo]	1.085 (0.887)	0.999 (0.182)	0.905
1418337565	6	keratin, type I cytoskeletal 10 isoform X2 [Canis lupus dingo]	1.085 (0.887)	0.999 (0.182)	0.905
1418337567	6	keratin, type I cytoskeletal 10 isoform X3 [Canis lupus dingo]	1.085 (0.887)	0.999 (0.182)	0.905
1418337649	3	puromycin-sensitive aminopeptidase isoform X1 [Canis lupus dingo]	0.523 (0.246)	1.047 (0.058)	0.100
1418337651	3	puromycin-sensitive aminopeptidase isoform X2 [Canis lupus dingo]	0.523 (0.246)	1.047 (0.058)	0.100
1418337655	3	puromycin-sensitive aminopeptidase isoform X4 [Canis lupus dingo]	0.523 (0.246)	1.047 (0.058)	0.100

1418338512	22	lactoperoxidase [Canis lupus dingo]	1.087 (1.969)	0.764 (0.019)	0.151
1418338550	4	keratin, type I cytoskeletal 15 [Canis lupus dingo]	0.4 (0.092)	1.009 (0.77)	0.200
1418338716	3	keratin, type I cytoskeletal 14 [Canis lupus dingo]	0.431 (0)	1.478 (0.844)	0.667
1418339572	3	keratin, type I cytoskeletal 24 [Canis lupus dingo]	0.28 (0)	2.241 (1.796)	0.667
1418341783	5	actin-related protein 2/3 complex subunit 2 [Canis lupus dingo]	0.906 (0.34)	0.86 (0.382)	0.548
1418342112	2	tubulin alpha-1B chain isoform X1 [Canis lupus dingo]	0.332 (0.664)	0.797 (0.12)	0.700
1418342114	2	tubulin alpha-1B chain isoform X2 [Canis lupus dingo]	0.332 (0.664)	0.797 (0.12)	0.700
1418342116	2	tubulin alpha-8 chain isoform X3 [Canis lupus dingo]	0.332 (0.664)	0.797 (0.12)	0.700
1418343247	4	fibronectin isoform X1 [Canis lupus dingo]	0.785 (0.703)	1.041 (0.501)	0.629
1418343249	4	fibronectin isoform X2 [Canis lupus dingo]	0.785 (0.703)	1.041 (0.501)	0.629
1418343251	4	fibronectin isoform X3 [Canis lupus dingo]	0.785 (0.703)	1.041 (0.501)	0.629
1418343253	5	fibronectin isoform X4 [Canis lupus dingo]	0.785 (0.703)	1.041 (0.501)	0.629
1418343255	5	fibronectin isoform X5 [Canis lupus dingo]	0.785 (0.703)	1.041 (0.501)	0.629
1418343257	4	fibronectin isoform X6 [Canis lupus dingo]	0.785 (0.703)	1.041 (0.501)	0.629
1418343259	5	fibronectin isoform X7 [Canis lupus dingo]	0.785 (0.703)	1.041 (0.501)	0.629
1418343261	4	fibronectin isoform X8 [Canis lupus dingo]	0.785 (0.703)	1.041 (0.501)	0.629
1418343263	5	fibronectin isoform X9 [Canis lupus dingo]	0.785 (0.703)	1.041 (0.501)	0.629
1418343265	4	fibronectin isoform X10 [Canis lupus dingo]	0.785 (0.703)	1.041 (0.501)	0.629
1418343267	5	fibronectin isoform X11 [Canis lupus dingo]	0.785 (0.703)	1.041 (0.501)	0.629
1418344784	10	heat shock protein HSP 90-alpha [Canis lupus dingo]	0.479 (0.251)	0.83 (0.097)	0.151
1418345245	6	L-lactate dehydrogenase A chain-like [Canis lupus dingo]	1.16 (0.692)	1.076 (0.535)	0.548
1418345461	3	proteasome subunit alpha type-3 [Canis lupus dingo]	0.303 (0)	0.887 (0.149)	0.667
1418345511	6	alpha-1-antitrypsin-like [Canis lupus dingo]	1.07 (1.216)	0.723 (0.536)	0.548
1418347612	5	alpha-actinin-1 isoform X1 [Canis lupus dingo]	0.809 (0.361)	1.001 (0.08)	0.095
1418347616	5	alpha-actinin-1 isoform X3 [Canis lupus dingo]	0.809 (0.361)	1.001 (0.08)	0.095
1418348746	2	NADP-dependent malic enzyme isoform X1 [Canis lupus dingo]	0.668 (0.31)	0.788 (0)	1.000
1418348750	2	NADP-dependent malic enzyme isoform X3 [Canis lupus dingo]	0.668 (0.31)	0.788 (0)	1.000
1418501820	4	rab GDP dissociation inhibitor beta isoform X1 [Canis lupus dingo]	0.809 (0.349)	0.983 (0.143)	0.841
1418501822	4	rab GDP dissociation inhibitor beta isoform X2 [Canis lupus dingo]	0.809 (0.349)	0.983 (0.143)	0.841
1418501824	4	rab GDP dissociation inhibitor beta isoform X3 [Canis lupus dingo]	0.809 (0.349)	0.983 (0.143)	0.841
1418503128	2	elongation factor 1-gamma [Canis lupus dingo]	0.76 (0.249)	0.895 (0.316)	0.310

1418503151	2	alpha-actinin-3 isoform X1 [Canis lupus dingo]	0.868 (0.033)	0.989 (0)	0.667
1418503153	2	alpha-actinin-3 isoform X2 [Canis lupus dingo]	0.868 (0.033)	0.989 (0)	0.667
1418503155	2	alpha-actinin-3 isoform X3 [Canis lupus dingo]	0.868 (0.033)	0.989 (0)	0.667
1418503443	3	cystatin-M [Canis lupus dingo]	1.164 (1.354)	0.968 (0.191)	0.222
1418504468	2	secretoglobin family 1D member 2-like [Canis lupus dingo]	0.503 (0.134)	0.317 (0.398)	0.905
1418505824	11	14-3-3 protein sigma [Canis lupus dingo]	0.793 (0.659)	1.079 (0.167)	0.690
1418506694	2	EF-hand domain-containing protein D2 [Canis lupus dingo]	0.543 (0.357)	0.913 (0.412)	0.229
1418507047	7	liver carboxylesterase 1 [Canis lupus dingo]	0.884 (0.401)	0.95 (0.537)	0.310
1418507405	2	protein-arginine deiminase type-4 [Canis lupus dingo]	0.733 (0.467)	1.091 (0.563)	0.095
1418507526	4	serine protease inhibitor Kazal-type 5 [Canis lupus dingo]	0.611 (0.143)	0.86 (0.254)	0.095
1418507824	2	F-actin-capping protein subunit beta isoform X3 [Canis lupus dingo]	0.501 (0)	0.925 (0.168)	0.667
1418509128	2	alpha-actinin-2 isoform X1 [Canis lupus dingo]	0.868 (0.033)	0.989 (0)	0.667
1418509130	2	alpha-actinin-2 isoform X2 [Canis lupus dingo]	0.868 (0.033)	0.989 (0)	0.667
1418510222	4	ceruloplasmin isoform X1 [Canis lupus dingo]	0.464 (0.347)	1.028 (0.081)	0.063
1418510226	4	ceruloplasmin isoform X2 [Canis lupus dingo]	0.464 (0.347)	1.028 (0.081)	0.063
1418510228	4	ceruloplasmin isoform X3 [Canis lupus dingo]	0.464 (0.347)	1.028 (0.081)	0.063
1418510230	4	ceruloplasmin isoform X4 [Canis lupus dingo]	0.464 (0.347)	1.028 (0.081)	0.063
1418510295	8	ceruloplasmin-like [Canis lupus dingo]	0.529 (0.568)	0.839 (0.23)	0.190
1418511015	7	14-3-3 protein zeta/delta-like [Canis lupus dingo]	0.867 (0.144)	1.01 (0.284)	0.548
1418511026	3	uridine phosphorylase 1 [Canis lupus dingo]	0.408 (0.46)	1.007 (0.136)	0.400
1418511817	6	superoxide dismutase [Cu-Zn] [Canis lupus dingo]	0.761 (0.605)	0.933 (0.152)	0.730
1418514065	2	proteasome subunit alpha type-4 [Canis lupus dingo]	0.583 (0)	0.912 (0.228)	0.667
1418514557	3	L-lactate dehydrogenase B chain-like [Canis lupus dingo]	0.914 (0.102)	0.77 (0.094)	0.114
1418514978	10	6-phosphogluconate dehydrogenase, decarboxylating [Canis lupus dingo]	1.037 (0.694)	0.739 (0.222)	0.730
1418515459	3	kininogen-1 isoform X1 [Canis lupus dingo]	0.613 (0.586)	0.911 (0.392)	0.400
1418515461	3	kininogen-1 isoform X2 [Canis lupus dingo]	0.613 (0.586)	0.911 (0.392)	0.400
1418515495	2	alpha-2-HS-glycoprotein [Canis lupus dingo]	1.242 (1.485)	0.959 (0.582)	1.000
1418515534	2	fetuin-B [Canis lupus dingo]	0.543 (0.645)	0.988 (0.527)	0.548
1473222592	3	protein S100-A4 isoform 2 [Canis lupus familiaris]	0.258 (0.607)	0.87 (0.304)	0.095
1494245195	2	immunoglobulin light chain IGL-1, partial [Canis lupus familiaris]	1.249 (0.652)	0.82 (0.444)	0.421
1494245199	2	immunoglobulin light chain IGL-3, partial [Canis lupus familiaris]	1.249 (0.652)	0.82 (0.444)	0.421

1494245207	2	immunoglobulin light chain IGL-7, partial [Canis lupus familiaris]	1.249 (0.652)	0.82 (0.444)	0.421
1494245209	7	immunoglobulin heavy chain IGH-1 [Canis lupus familiaris]	0.814 (0.806)	0.843 (0.073)	1.000
1494245213	5	immunoglobulin heavy chain IGH-3 [Canis lupus familiaris]	0.755 (0.469)	0.724 (0.125)	0.690
1494245215	6	immunoglobulin heavy chain IGH-4 [Canis lupus familiaris]	0.68 (0.797)	0.813 (0.074)	1.000
1494245221	5	immunoglobulin heavy chain IGH-7 [Canis lupus familiaris]	0.755 (0.469)	0.724 (0.125)	0.690
1494245225	5	immunoglobulin heavy chain IGH-9 [Canis lupus familiaris]	0.755 (0.469)	0.724 (0.125)	0.690
1494245227	5	immunoglobulin heavy chain IGH-10 [Canis lupus familiaris]	0.755 (0.469)	0.724 (0.125)	0.690
1494245229	7	immunoglobulin heavy chain IGH-11 [Canis lupus familiaris]	0.732 (0.806)	0.823 (0.073)	1.000
1494245231	5	immunoglobulin heavy chain IGH-12 [Canis lupus familiaris]	0.755 (0.469)	0.724 (0.125)	0.690
1494245233	7	immunoglobulin heavy chain IGH-13 [Canis lupus familiaris]	0.732 (0.806)	0.823 (0.073)	1.000
1494245235	5	immunoglobulin heavy chain IGH-14 [Canis lupus familiaris]	0.755 (0.469)	0.724 (0.125)	0.690
1494245237	5	immunoglobulin heavy chain IGH-15 [Canis lupus familiaris]	0.755 (0.469)	0.724 (0.125)	0.690
1494245239	5	immunoglobulin heavy chain IGH-16 [Canis lupus familiaris]	0.755 (0.469)	0.724 (0.125)	0.690
1494245241	5	immunoglobulin heavy chain IGH-17 [Canis lupus familiaris]	0.755 (0.469)	0.724 (0.125)	0.690

IQR: interquartile range.
